# Reproductive prognosis factors in women with infertility and polycystic ovary syndrome undergoing *in vitro* fertilization techniques

**DOI:** 10.5935/1518-0557.20250040

**Published:** 2025

**Authors:** Margarida Pinto Ribeiro, Carolina Lemos, Carla Leal, Márcia Barreiro, António Tomé, Emídio Vale-Fernandes

**Affiliations:** 1 ICBAS - School of Medicine and Biomedical Sciences, UMIB - Unit for Multidisciplinary Research in Biomedicine, University of Porto, Porto, Portugal; 2 ITR - Laboratory for Integrative and Translational Research in Population Health, University of Porto, Porto, Portugal; 3 Centro de Procriação Medicamente Assistida / Banco Público de Gâmetas, Centro Materno-Infantil do Norte Dr. Albino Aroso (CMIN), Centro Hospitalar Universitário de Santo António (CHUdSA), Unidade Local de Saúde de Santo António (ULSSA), Porto, Portugal; 4 Departamento da Mulher e da Medicina Reprodutiva, Centro Materno-Infantil do Norte Dr. Albino Aroso (CMIN), Centro Hospitalar Universitário de Santo António (CHUdSA), Unidade Local de Saúde de Santo António (ULSSA), Porto, Portugal

**Keywords:** polycystic ovary syndrome, infertility, *In vitro* fertilization/intracytoplasmatic sperm injection, body mass index, anti-Müllerian hormone

## Abstract

**Objective:**

Polycystic ovary syndrome (PCOS) is the most prevalent endocrinopathy and is often associated with elevated levels of anti-Müllerian hormone (AMH) and obesity. AMH may influence the reproductive prognosis of women undergoing in vitro fertilization (IVF). This study aims to explore fundamental reproductive characteristics and intra-cycle variables related to IVF and their association with reproductive success in women with PCOS.

**Methods:**

This retrospective study involved 393 women with PCOS who underwent IVF. It was designed to evaluate the relationship between AMH levels, body mass index (BMI), age, and the luteinizing hormone/follicle-stimulating hormone (LH:FSH) ratio, along with reproductive outcomes. The sample was categorized based on AMH percentiles, BMI classes, age, and LH:FSH ratio.

**Results:**

A negative correlation was observed between AMH levels and age. There were no significant differences in BMI across AMH groups, except in the very high AMH group, where women were found to be overweight. The LH:FSH ratio increased with AMH levels. Notably, women over the age of 35 with elevated AMH levels exhibited lower live birth rates (LBR) and cumulative live birth rates (CLBR) compared to their younger counterparts within the same AMH percentile range. A decrease in the fertilization rate was noted among overweight women.

**Conclusions:**

While AMH measurement may assist in clinical decision-making, it should not be regarded as a sole predictor of IVF success. Age appears to have a more substantial impact on LBR compared to BMI. In practice, CLBR is the most informative metric to convey reproductive success to couples. Variations in results among studies could be attributed to differences in populations, sample sizes, and inconsistencies in the definitions of LBR and CLBR.

## INTRODUCTION

Polycystic ovary syndrome (PCOS) is a significant cause of oligomenorrhea and hyperandrogenism in women and can be readily diagnosed when classic signs and symptoms, such as hirsutism and polycystic ovarian morphology, are present (Joham *et al*., 2022). PCOS is the most prevalent endocrinopathy, affecting approximately 5 to 18% of women of reproductive age (Joham *et al*., 2022). Its prevalence is often linked to various metabolic disorders, including infertility, obesity, diabetes mellitus, and an increased risk of long-term pregnancy complications and cardiovascular diseases (Solomon *et al*., 2002; Moran *et al*., 2010; Wild *et al*., 2011; Diamanti-Kandarakis & Dunaif, 2012; Christ *et al*., 2019). There is considerable heterogeneity in the phenotypic manifestations of this syndrome, and the consequences can vary significantly throughout a woman’s life (Clark *et al*., 2014; van Keizerswaard *et al*., 2022).

In PCOS, obesity is a common characteristic, estimated to affect approximately half of the women with this condition, with the increase in fat mass often coinciding with the onset of PCOS. However, obesity is not part of the diagnostic criteria, as about 20% of women with PCOS are not obese. Hormonal studies in women with PCOS have revealed elevated levels of testosterone and estrogen, demonstrating an increased ratio of luteinizing hormone (LH) to follicle-stimulating hormone (FSH), as well as higher concentrations of estrone compared to estradiol, and androstenedione levels that are at the upper limits of normal or elevated (Casanova *et al*., 2019).

Currently, the most widely accepted diagnostic criteria for PCOS are the Rotterdam criteria, which require the presence of at least two of the following three parameters: menstrual cycle irregularities, clinical or biochemical hyperandrogenism, and polycystic ovarian morphology (Christ & Cedars, 2023). This diagnostic method is subject to controversy in the medical community because some classical characteristics are not always present (Clark *et al*., 2014). In 2012, the National Institute of Health recommended the use of this classification, identifying specific subtypes for PCOS (NIH, 2012). Several laboratories, clinical, and physical parameters can be evaluated in PCOS, including total and free testosterone hormone levels, dehydroepiandrosterone sulfate, androstenedione, 17-hydroxyprogesterone (Longcope, 1986; Burger, 2002) and anti-Müllerian hormone (AMH), which are not included in the Rotterdam criteria. Due to the heterogeneity associated with ultrasound evaluation in PCOS, there has been growing interest in using AMH as a possible surrogate marker for ovarian morphology assessment (Dumont *et al*., 2015), because it is typically elevated in women with PCOS compared to normo-ovulatory women (Cook *et al*., 2002).

According to international guidelines for the assessment and treatment of PCOS, anovulatory infertility can be effectively managed with first-line therapies, including ovulation induction using either monotherapy or a combination of medications. For those who do not respond to first-line treatments, second-line therapy may involve ovarian stimulation in conjunction with assisted reproductive techniques, such as *in vitro* fertilization (IVF) (Teede *et al*., 2018). Despite the most of women with PCOS responding favorably to ovarian stimulation for IVF, with a higher than average number of growing follicles, their reproductive outcomes tend to be unfavorable compared to women with infertility due to other causes (Rajani *et al*., 2012; Piomboni *et al*., 2014; Yilmaz *et al*., 2016; Artimani *et al*., 2018). Factors related to oocyte quality (Qiao & Feng, 2011) and endometrial competence (Piltonen, 2016) may negatively impact reproductive outcomes, suggesting that this condition could deteriorate the follicular microenvironment and compromise the physiological functions of oocytes (Palomba *et al*., 2017).

AMH is a polypeptide secreted by the granulosa cells of preantral and small antral ovarian follicles (van Rooij *et al*., 2002), and it is widely recognized as a sensitive marker of ovarian reserve (La Marca *et al*., 2010; Tal *et al*., 2014). This hormone plays a crucial role in inhibiting the activity of primary follicles and regulating their growth and development (Chang *et al*., 2019). AMH levels are significantly elevated in women with PCOS, and numerous studies have sought to establish a correlation between AMH levels and the morphological characteristics of polycystic ovaries (Swanson *et al*., 1981; Reed & Carr, 2000; Slayden *et al*., 2001; DeUgarte *et al*., 2006; Escobar-Morreale *et al*., 2012; Legro *et al*., 2013; Prior *et al*., 2015; Bull *et al*., 2019; Carmina *et al*., 2019; Starace *et al*., 2020). Additionally, AMH has been considered a predictor of ovarian response to controlled ovarian stimulation (Delamuta *et al*., 2024). It is known that both AMH levels and the number of ovarian follicles decline as a woman age, occurring in both women with and without PCOS (Erdem *et al*., 2002; Alsamarai *et al*., 2009; de Kat *et al*., 2016). This observation has led some researchers to suggest the necessity of age-adjusted diagnostic criteria. While specific age-related thresholds have yet to be widely accepted by the scientific community, recognizing these changes over time is important, particularly when assessing older women with PCOS (Christ & Cedars, 2023). Although exploring this relationship holds promise, considerable uncertainty remains regarding the methodologies used in studies and the ranges of AMH levels that have been investigated. Due to these limitations, AMH is not included in current guidelines as a substitute for imaging evaluations of polycystic ovaries or as a standalone test for diagnosing PCOS (Teede *et al*., 2018).

Originally, it was believed that the elevated levels of AMH in individuals with PCOS resulted from the presence of polycystic ovaries, which contain approximately twice as many preantral and antral follicles. However, recent insights indicate that increased AMH levels are primarily due to enhanced production by individual follicles (Das *et al*., 2008) and may also correlate with the severity of PCOS (La Marca *et al.*, 2004a; Nardo *et al.*, 2009; Piouka *et al.*, 2009; Lin *et al.*, 2011; Delamuta *et al*., 2024). Multiple recent studies have demonstrated that AMH possesses significant predictive validity in diagnosing PCOS, particularly concerning the severity of ovulatory dysfunction (La Marca *et al*., 2010). Therefore, AMH could serve as an important indicator in assessing the reproductive prognosis of women with PCOS who are undergoing assisted reproductive technologies (ART) (Vale-Fernandes *et al*., 2023). While still a matter of debate, the latest evidence suggests that AMH levels may also be associated with reproductive outcomes in ART, including pregnancy rates and live birth rates (LBR), independent of age (Eldar-Geva *et al*., 2005; Nelson *et al*., 2007; Elgindy *et al*., 2008; Gleicher *et al*., 2010; Blazar *et al*., 2011; La Marca *et al*., 2011; Brodin *et al*., 2013), as well as with a higher yield of mature oocytes and embryos (Fallat *et al*., 1997; Hazout *et al*., 2004; La Marca *et al*., 2004b; Eldar-Gevat *et al*., 2005; Lekamge *et al*., 2007). A recent study also suggested that AMH levels could guide the selection of ovarian stimulation protocols, with the aim of improving treatment outcomes for women of advanced reproductive age (Meczekalski *et al*., 2016a; 2016b). However, conflicting data exist regarding the effectiveness of AMH as a prognostic marker in IVF techniques, leading to its status as a tool not universally accepted (Kaya *et al*., 2010; Tal *et al*., 2014; Liu *et al*., 2022).

Anovulatory infertility is often the first sign of PCOS (Cunha & Póvoa, 2021). When first-line treatments prove ineffective, these women may pursue second-line infertility therapies, such as in IVF or intracytoplasmic sperm injection (ICSI) (Costello *et al*., 2019). In recent years, some reproductive prognostic factors have been identified as predictors of success after ART (Gao *et al*., 2021; Wang *et al*., 2023). These factors include age, body mass index (BMI), baseline concentrations of AMH and follicle-stimulating hormone (FSH) at the start of the menstrual cycle, and follicle count (Tsakos *et al*., 2014). This study was designed to clarify the prognostic value of AMH levels, age, BMI, and the LH:FSH ratio in women with PCOS undergoing IVF/ICSI techniques.

## MATERIAL

### Study design and participants

This study is an original article based on a retrospective observational analysis involving a sample of women with PCOS (n=393) who underwent IVF/ICSI using autologous oocytes at the Centro Materno-Infantil do Norte (CMIN), Porto, Portugal. The participants were monitored from the initial study phase through to the completion of reproductive techniques, pregnancy, and childbirth. Data were collected from each oocyte pick-up (n=494) between January 2017 and December 2022, with follow-up on pregnancies and births conducted from the date of the initial embryo transfer until December 2023. The study utilized the current Rotterdam diagnostic criteria for participant inclusion (Christ & Cedars, 2023). Cases presumed to be PCOS but lacking the requisite signs and symptoms outlined in the Rotterdam criteria, as well as those exhibiting clinical or biochemical hyperandrogenism due to other causes, were excluded. Additionally, cases involving endometriosis, thyroid disease, and other endocrine disorders were also excluded. Electronic clinical records were reviewed to extract the necessary data for the research. The study protocol received approval from the Ethics Committee of the Unidade Local de Saúde de Santo António (ULSSA) on February 1, 2024, under the Reference Number: 2023.262(224-DEFI/214-CE).

### Sample division by levels of AMH

The sample was divided according to serum levels of AMH: below the 25th percentile (AMH <1.40 ng/mL; n=88), between the 25th and 75th percentiles (AMH between 1.40 and 4.67 ng/mL; n=172) and above the 75th percentile (AMH >4.67 ng/mL; n=86). The terms “low”, “moderate”, “high”, and “very high” correspond to the results relative to the groups below the 25th percentile, between the 25th and 75th percentiles, above the 75th percentile, and above the 95th percentile, respectively, and are mentioned to simplify the analysis and discussion of results, not necessarily indicating reference values or biological normality. A more detailed analysis was performed in the subgroup of women with AMH levels above the 95th percentile (AMH >8.67ng/mL; n=17).

### Sample division by age

The sample was divided categorizing the sample into two groups according to the age of the female subject: below 35 years (n=238) and above 35 years (n=256).

### Sample division by BMI classes

The sample was divided categorizing the sample into BMI classes: underweight (BMI <18.5 kg/m^2^; n=23), normal weight (BMI between 18.5 and 24.9 kg/m^2^; n=285), overweight (BMI between 25.0 and 29.9 kg/m^2^; n=106), and obesity (BMI ≥30.0 kg/m^2^; n=79).

### Sample division by LH:FSH ratio

The sample was divided categorizing the sample into two categories of the LH:FSH ratio, below (n=305) and above 1 (n=178).

### Ovarian stimulation and IVF procedures

The women underwent an antagonist protocol of gonadotropin-releasing hormone (GnRH) with controlled and individualized ovarian stimulation, based on standard tests of ovarian reserve. On the third day after oocyte pick up, one or two embryos were selected for transfer, while the remaining embryos were cryopreserved or allowed to develop until the blastocyst stage for fresh embryo transfer or to be frozen for subsequent frozen embryo transfer (FET). The decision to freeze all embryos was made based on local criteria, which included factors such as the occurrence or risk of ovarian hyperstimulation syndrome, inadequate endometrial environment, premature elevation of progesterone, COVID-19 infection, hydrosalpinx, or personal circumstances favoring FET rather than fresh embryo transfer.

### Primary and secondary assessment measures

The primary outcomes included LBR and the cumulative live birth rate (CLBR). Live birth was defined as the birth of one or more live infants. LBR was defined as the number of live births divided by the number of women in a group. CLBR was defined as the rate of live births that occurred during the fresh IVF cycle and subsequent FET cycle(s) after a single oocyte pick-up until achieving a live birth. Clinical pregnancy was defined as the visualization of one or more gestational sacs on ultrasound. Pregnancies without visualization of a gestational sac and/or without visualization of a gestational sac with a heartbeat were considered miscarriages and did not contribute to clinical pregnancy rate. The cumulative pregnancy rate was defined as the proportion of women who became pregnant from a single oocyte pick-up.

The secondary outcomes included the rate of oocyte immaturity, defined as the proportion of immature oocytes per number of cumulus-oocyte complexes (COC); fertilization rate, defined as the proportion of embryos with two pronucleated oocytes (2PN) per number of COC; cleavage rate, defined as the proportion of the number of 2PN embryos by the number of 2PN; blastocyst rate, defined as the proportion of the number of blastocysts by the number of 2PN; the percentage of freeze-all cycles and the number of embryos obtained and cryopreserved.

### Statistical analysis

The primary and secondary statistical analysis involved comparing group characteristics, FIV/ICSI cycles, and obstetric outcomes. Categorical variables were presented as number of cases and percentages, and continuous variables as mean, standard deviation, minimum, and maximum.

For categorical variables in group comparisons, the Chi-square test was used, and for continuous variables, the One-Way ANOVA test was used. The degree of association of AMH levels, age, BMI, and LH:FSH ratio with reproductive prognostic factors was calculated using the Spearman correlation coefficient. Logistic regression was performed to verify age as a confounder in the analysis by percentiles of AMH groups.

Data from this study were analyzed using IBM SPSS Statistics 29.0.0.0 Software. A statistical significance level of 0.05 (5%) was considered.

## RESULTS

The sample of women with PCOS in this study has a mean age of 34.78 years (±3.55), a mean BMI of 24.76 kg/m^2^ (±5.12), and a mean AMH level of 3.38 ng/mL (±2.77). At the beginning of the menstrual cycle, the women in the sample had a serum LH level of 7.20mIU/mL (.81), FSH level of 7.67mIU/mL (±5.51), basal estradiol level of 49.33mIU/mL (±41.78), and the LH:FSH ratio was 1.02 (±0.66). According to the technique, 52.2% underwent ICSI and 47.8% underwent IVF. Regarding smoking, 21.2% of women reported this habit, while 40.3% of male partners too. Only 9.9% of women had other causes of infertility besides PCOS, and 66.4% had concomitant male infertility. Additional results can be found in Table 1.

**Table 1:** Descriptive analysis of the sample.

	Mean	Standard deviation		Minimum	Maximum		n
Age (years)	34.78	3.55		22.00	40.00		494
Weight (Kg)	65.70	13.67		42.00	130.00		493
Hight (cm)	162.97	6.09		137.00	181.00		494
BMI (Kg/m^2^)	24.76	5.12		16.70	52.70		493
Duration of infertility (months)	51.16	32.49		0.00	252.00		493
AMH. ng/dL	3.38	2.77		0.05	20.41		346
LH.mlU/mL	7.20	4.81		1.70	74.70		483
FSH.mlU/mL	7.67	5.51		2.20	112.00		486
LH:FSH ratio	1.02	0.66		0.29	8.96		483
Estradiol. pg/mL	49.33	41.78		3.00	503.00		464
Clinical pregnancy rate (%)	38.49	46.39		0.00	100.00		422
Cumulative clinical pregnancy rate (%)	43.93	46.69		0.00	100.00		428
LBR (%)	32.87	45.14		0.00	100.00		416
CLBR (%)	37.44	48.45		0.00	100.00		422
Endometrial thickness on the day of the last monitoring (mm)	9.88	2.04		0.00	18.32		482
Endometrial thickness on oocyte pick-up day (mm)	10.47	2.22		5.90	19.00		166
			%			n	
Primary infertility type			78.50			493	
Female smoker [n (%)]			21.20			217	
Male smoker [n (%)]			40.30			216	
Couple smoker [n (%)]			15.30			216	
PCOS + other infertility factor [n (%)]			9.90			494	
Male infertility factor [n (%)]			66.40			494	
ICSI			52.20			494	
FIV			47.80			494	

BMI: body mass index; AMH: anti-Müllerian hormone; LH: luteinizing hormone (at beginning of the menstrual cycle); FSH: follicle-stimulating hormone (at beginning of the menstrual cycle); PCOS: polycystic ovary syndrome; IU - International units; ICSI: Intracytoplasmatic sperm injection; FIV: *in vitro* fertilization.

### According to Percentiles of AMH

The IVF/ICSI cycles (n=494) were categorized into groups based on AMH levels (n=346) (Table 2). Women with PCOS and low AMH levels tended to be older (*p*<0.001). When comparing women with high and low AMH levels, those in the high AMH group exhibited significantly lower FSH levels (6.50 *vs*. 10.37/mL; *p*<0.001), a higher LH:FSH ratio (1.37 *vs*. 0.68; *p*<0.001), and higher LH levels (8.35 *vs*. 6.92mIU/mL), although the difference in LH levels was not statistically significant. No significant differences in BMI were observed between the groups (25.33 *vs*. 24.25 *vs*. 25.68 kg/m^2^). However, when examining the subgroup of women with AMH levels above the 95th percentile (n=17), it was found that they tended to have a higher weight (73.59 kg; *p*=0.020) and a higher BMI (28.09 kg/m^2^; *p*=0.009) compared to those below the 95th percentile. Additionally, this group of women with very high AMH levels exhibited a continued trend of an elevated LH:FSH ratio (*p*<0.001).

**Table 2:** Baseline reproductive characteristics and hormone levels of *in vitro* fertilization (IVF) cycles according to the anti-Müllerian hormone (AMH) levels (n=346).

	Low (<1,40 ng/mL) (n=88)	Moderate (1,40-4,66 ng/mL) (n=172)	High (>4,66 ng/mL) (n=86)	p-value	Very high (>8,67 ng/mL) (n=17)	p-value
Age (years)	36.17±3.35	34.6613.28	33.6413.63	<0.001^ab^	33.2414.31	NS^d^
Weight (Kg)	67.89±15.06	64.04112.56	67.47115.31	NS^a^; NS^b^	73.59114.20	0.020^d^
Height (cm)	163.75±5.86	162.4615.77	162.1516.10	NS^a^; NS^b^	162.0018.07	NS^d^
BMI (Kg/m^2^)	25.33±5.69	24.2514.55	25.6815.73	NS^a^; NS^b^	28.0915.55	0.009^d^
Primary infertility type [n (%)]	68 (78.2)	136 (79.1)	69 (80.2)	NS^C^	13 (76.5)	NS^C^
Female smoker [n (%)]	7(8.0)	16 (9.3)	14 (16.3)	NS^C^	2 (11.9)	NS^C^
Male smoker [n (%)]	17 (19.3)	34(19.8)	22 (25.6)	NS^C^	4(23.5)	NS^C^
Couple smoker [n (%)]	6(13.6)	9(9.7)	10 (21.7)	NS^C^	2 (18.2)	NS^C^
PCOS+other infertility factor [n (%)]	9 (10.2)	23 (13.4)	9 (10.5)	NS^C^	3(17.6)	NS^C^
Male infertility factor [n (%)]	61 (69.3)	121 (70.3)	61 (70.9)	NS^C^	11 (64.7)	NS^C^
Duration of infertility (months)	49.84±34.94	53.39130.78	49.95131.66	NS^a^; NS^b^	43.06118.52	NS^d^
LH,mlU/mL	6.92±8.11	7.0813.23	8.3515.12	NS^a^; NS^b^	8.5113.46	NS^d^
FSH,mlU/mL	10.37±11.78	7.3612.31	6.5011.71	<0.001^a^; NS^b^	5.8011.91	NS^d^
LH:FSH ratio	0.68±0.23	1.0210.53	1.3711.08	<0.001^ab^	1.5910.84	<0.001^d^
Basal estradiol, pg/mL	51.37±39.92	48.24135.55	50.81144.72	NS^a^; NS^b^	47.92120.58	NS^d^
Progesterone, ng/mL	1.0411.92	0.9110.60	1.5010.95	NS^a^; NS^b^	-	___

Data expressed as mean±standard deviation. *p*-value < 0.05 was considered statistically significant. BMI: body mass index; PCOS: polycystic ovary syndrome; LH: luteinizing hormone (at beginning of the menstrual cycle); FSH: follicle-stimulating hormone (at beginning of the menstrual cycle); IU - International units; Pc - Percentile; NS - not statistically different; a - Comparison between “low” and “high” (One Way ANOVA); b - Comparison between “moderate” and “high” (One Way ANOVA); c - Chi-square test; d - Comparison between “very high” and below “very high” (One Way ANOVA).

The IVF cycle characteristics and outcomes are presented in Table 3. The gonadotropin dose used in stimulation was lower in the high AMH group compared to the low AMH group (2082.06 IU *vs*. 2658.91 IU; *p*<0.001). The number of follicle count (5.46 *vs*. 13.29 *vs*. 21.58; *p*<0.001 and *p*<0.001), the number of COC (6.35 *vs*. 11.22 *vs*. 13.91; *p*<0.001; *p*=0.015), and embryos with 2PN (3.12 *vs*. 5.04 *vs*. 6.33; *p*<0.001; *p*=0.033) were higher in the high AMH group compared to the low and moderate AMH groups. Women with low AMH levels obtained fewer embryos compared to the high AMH group (1.73 *vs*. 2.73; *p*<0.001), with no statistically significant differences between the moderate and high AMH groups. Across AMH percentiles, there is a statistically significant trend of increasing number of freeze all IVF cycles between groups (23.9% *vs*. 47.7% *vs*. 61.6%; *p*<0.001). It is also noted that the clinical pregnancy rate and cumulative pregnancy rate increase across AMH percentiles; however, these differences between groups are not statistically significant. Regarding biochemical pregnancy, this factor tends to be higher with higher AMH levels, with statistical significance between groups (35.2% *vs*. 52.3% *vs*. 53.4%; *p*=0.004). As for LBR, there are significant differences between AMH groups, with an increase between the low and moderate AMH group (21.7% *vs*. 33.8%), but a slight decrease between the moderate and high AMH group (33.8% *vs*. 32.4%; *p*=0.015). The CLBR increased across AMH percentiles (22.4% *vs*. 39.3% *vs*. 41.4%; *p*=0.021), with statistically significant differences between groups. There were no statistically significant results for the number of stimulation days, nor for fertilization rates, embryo cleavage rates, blastocyst rates, and endometrial thickness on the day of final monitoring and on the day of oocyte pick-up. The remaining results are shown in Tables 2 and 3.

**Table 3:** In vitro fertilization (IVF) intra-cycle characteristics and assisted reproductive technology (ART) outcomes according to the anti-Müllerian hormone (AMH) levels (n=346).

	Low (<1.40 ng/mL) (n=88)	Moderate (1.40-4.66ng/mL) (n=172)	High (>4.66ng/mL) (n=86)	p-value	Very high (>8.67 ng/ml) (n=17)	p-value
ICSI (%)	49 (55.7)	94 (54.6)	51 (59.3)	NS^C^	6(35.3)	NS^C^
Gonadotropin dose (IU)	2658.91±1187.26	2461.86±837.60	2082.06±681.94	<0.001^a^; NS^b^	2194.12±761.09	NS^d^
Number of stimulation days	10.03±2.62	9.90±1.90	9.96±1.86	NS^a^; NS^b^	10.65±2.12	NS^d^
Number of follicles	5.46±2.49	13.29±9.08	21.58±10.58	<0.001^ab^	27.14±7.56	<0.001^d^
Number of COC	6.35±5.54	11.22±7.74	13.91±7.82	<0.001^a^; 0.015^b^	15.24±12.20	0.012^d^
Oocyte immaturity rate (%)	18.52±18.66	18.22±19.05	22.48±20. 07	NS^a^; NS^b^	32.56±23.24	0.006^d^
Number of 2PN oocytes	3.12±2.43	5.04±4.00	6.33±4.51	<0.001^a^; 0.033^b^	6.88±5.07	0.032^d^
Fertilization rate (%)	51.4	50.1	45.38	NS^a^; NS^b^	48.24	NS^d^
Cleavage rate (%)	98.5	96.73	98.37	NS^a^; NS^b^	99.58	NS^d^
Blastocyst rate (%)	8.05	7.75	9.53	NS^a^; NS^b^	2.50	NS^d^
Number of obtained embryos	1.73±1.09	2.54±1.80	2.73±2.02	<0.001^a^; NS^b^	3.18±2.24	NS^d^
Number of IVF cycles with *freeze all* [n (%)]	21 (23.9)	82 (47.7)	53 (61.6)	<0.001^c^	12 (70.6)	0.030^c^
Number of criopreserved embryos	0.66±1.08	1.73±1.97	2.28±2.38	<0.001^a^; NS^b^	3.29±2.49	0.002^d^
Positive βHCG [n (%)]	31 (35.2)	90 (52.3)	46(53.4)	0.004^c^	10 (66.7)	NS^C^
Clinical pregnancy rate (%)	30.3	39.0	39.0	NS^C^	35.6	<0.001^c^
Cumulative clinical pregnancy (%)	33.8	45.7	48.6	NS^C^	53.3	NS^C^
LBR (%)	21.7	33.8	32.4	0.015^c^	20.0	<0.001^c^
CLBR (%)	22.4	39.3	41.4	0.021^c^	33.3	NS^C^
Endometrial thickness on the day of the last monitoring (mm)	9.66±1.87	9.98±1.91	10.24±2.40	NS^a^; NS^b^	10.83±2.41	NS^d^
Endometrial thickness on oocyte pick-up day (mm)	9.91±1.95	10.47±2.30	10.97±2.28	NS^a^; NS^b^	11.07±2.11	NS^d^

Data expressed as mean±standard deviation. *p*-value < 0.05 was considered statistically significant. ICSI - Intracytoplasmatic sperm injection; IU - International units; COC - cumulus-oocyte complexes; 2PN - two pronucleated; IVF - *in vitro* fertilization; βHCG - human chorionic gonadotropin hormone; LBR - live birth rate; CLBR - Cumulative live birth rate; Pc - Percentile; NS - not statistically different; a - Comparison between “low” and “high” (One Way ANOVA); b - Comparison between “moderate” and “high” (One Way ANOVA); c - Chi-square test; d - Comparison between “very high” and below “very high” (One Way ANOVA).

Conducting a logistic regression analysis for age as a potential confounding factor in the AMH groups, it is observed that, considering the same reproductive prognostic variables, with the sample divided into two groups of women below and above 35 years old, respectively, differences exist (Tables 4 and 5). In the group of women below 35 years old, the LH:FSH ratio was significantly higher between the high AMH group and the moderate AMH group (1.55 *vs*. 1.12; *p*<0.001). The dose of gonadotropins used in stimulation became statistically significant, with higher dose in the low AMH group (*p*<0.001). And the number of embryos obtained continued to trend upwards across groups but ceased to be statistically significant in this age group. The number of cryopreserved embryos was significantly higher in the high AMH group compared to the moderate AMH group and no longer showed statistically significant differences between the low and high AMH groups. Women below 35 years old in the high AMH group had higher progesterone levels at trigger (1.38 *vs*. 0.94 ng/mL; *p*=0.044) and a higher number of freeze-all IVF cycles (72.5% *vs*. 44.8%; *p*=0.002) compared to the moderate AMH group. The LBR and CLBR increased with AMH levels in younger women; however, without statistical significance between groups.

**Table 4:** Baseline reproductive characteristics and hormone levels of *in vitro* fertilization (IVF) cycles according to the anti-Müllerian hormone (AMH) levels in women below and above 35 years old (n=166; n=180).

	< 35 years						> 35 years					
	Low (<1.40ng/mL) (n=28)	Moderate (1.40-4.66ng/mL) (n=87)	High (>4.6ng/mL) (n=51)	p-value	Very high (>8.67ng/mL) (n=10)	p-value	Low (<1.40 ng/mL) (n=60)	Moderate (1.40-4.66ng/mL) (n=85)	High (>4.66ng/mL) (n=35)	p-value	Very high (>8.67ng/mL) (n=7)	p-value
Age (years)	32.14±3.04	31.93+2.23	31.25±2.70	NS^ab^	30.80+4.05	NS^d^	38.05±1.00	37.46il.ll	37.llil.13	0.004^a^;<0.001^b^	37.71i0.95	0.036^d^
Weight (Kg)	69.50±13.55	64.95+13.17	68.48±15.47	NS^ab^	73.60+10.98	NS^d^	67.13±15.76	63.llill.92	66.00il5.19	NS^ab^	73.57il8.89	NS^d^
Height (cm)	164.61±6.13	162.37±5.58	163.35±6.29	NS^ab^	164.80±9.09	NS^d^	163.35±5.75	162.55i5.99	160.40i5.44	NS^ab^	158.00i4.24	0.043^d^
BMI (Kg/m^2^)	25.66±4.87	24.58+4.56	25.61±5.22	NS^ab^	27.18+4.11	NS^d^	25.18±6.06	23.91i4.55	25.79i6.49	NS^ab^	29.40i7.31	0.021^d^
Primary infertility type [n (%)]	26 (92.9)	72 (82.8)	45 (88.2)	NS^C^	9 (90.0)	NS^;^	42 (70.0)	64 (75.3)	24 (68.6)	NS^C^	4(57.1)	NS^C^
Female smoker [n (%)]	2(13.3)	4 (10.0)	10 (37.0)	0.015^c^	2 (20.0)	NS^c^	5 (8.3)	12 (14.1)	4 (11.4)	NS^C^	0 (0.0)	NS^C^
Male smoker [n (%)]	5 (33.3)	12 (30.0)	16 (61.5)	0.027^c^	3 (30.0)	NS^C^	12 (20.0)	22 (25.9)	6(17.1)	NS^C^	1 (14.3)	NS^C^
Couple smoker [n (%)]	2(13.3)	3 (7.5)	8 (30.8)	0.040^c^	2 (20.0)	NS^C^	4(6.7)	6(7.1)	2 (5.7)	NS^C^	0 (0.0)	NS^C^
PCOS + other infertility factor [n (%)]	5 (17.9)	8(9.2)	3 (5.9)	NS^C^	0 (0.0)	NS^C^	4(6.7)	15 (17.6)	6(17.1)	NS^C^	3 (42.9)	0.024^c^
Male infertility factor [n (%)]	21 (75.0)	77 (88.5)	41 (80.4)	NS^C^	9 (90.0)	NS^C^	40 (66.7)	44 (51.8)	20 (57.1)	NS^C^	2 (28.6)	NS^C^
Duration of infertility (months)	49.71±27.95	48.61±28.31	47.88±24.13	NS^ab^	43.20+21.87	NS^d^	49.90i37.99	58.24i32.55	52.97i40.42	NS^ab^	42.86il4.04	NS^d^
LH,mlU/mL	5.69±2.17	7.56±3.40	8.92±6.19	NS^a^; 0.006^b^	8.89+4.21	NS^d^	7.49i9.68	6.61±2.99	7.52i2.88	NS^ab^	7.87±1.80	NS^d^
FSH,mlU/mL	8.66±3.10	7.22±2.15	6.29±1.75	0.010^a^; <0.001^b^	5.90+2.39	NS^d^	ll.18il4.ll	7.49i2.45	6.82±1.62	0.042^a^; 0.026^b^	5.63i0.81	NS^d^
LH:FSH ratio	0.69±0.19	1.12±0.60	1.55±1.35	NS^a^; <0.001^b^	1.71+1.06	NS^d^	0.68i0.25	0.91±0.41	1.10±0.36	<0.001^ab^	1.39i0.21	<0.001^d^
Basal estradiol, pg/mL	45.16±19.49	48.70+45.70	56.24±54.59	NS^ab^	43.20±10.84	NS^d^	54.16i46.14	47.76i21.09	43.03i23.27	NS^ab^	58.52i34.ll	NS^d^
Progesterone, ng/mL	0.55±0.34	0.94±0.58	1.38±1.14	NS^a^; 0.044^b^	...	...	1.28i2.32	0.88i0.64	1.71±0.53	NS^ab^	...	...

Data expressed as mean±standard deviation. *p*-value < 0.05 was considered statistically significant. BMI: body mass index; PCOS: polycystic ovary syndrome; LH: luteinizing hormone (at beginning of the menstrual cycle); FSH: follicle-stimulating hormone (at beginning of the menstrual cycle); IU - International units; Pc - Percentile; NS - not statistically different; a - Comparison between “low” and “high” (One Way ANOVA); b - Comparison between “moderate” and “high” (One Way ANOVA); c - Chi-square test; d - Comparison between “very high” and below “very high" (One Way ANOVA).

**Table 5:** In vitro fertilization (IVF) intra-cycle characteristics and assisted reproductive technology (ART) outcomes according to the anti-Müllerian hormone (AMH) levels in women below and above 35 years old (n=166; n=180).

	< 35 years						> 35 years					
	Low (<1.40 ng/mL) (n=28)	Moderate (1.40-4.66ng/mL) (n=87)	High (>4.66 ng/mL) (n=51)	p-value	Very high (>8.67 ng/ml) (n=10)	p-value	Low (<1.40 ng/mL) (n=60)	Moderate (1.40-4.66 ng/mL) (n=85)	High (> 4.66 ng/mL) (n=35)	p-value	Very high (>8.67 ng/ml) (n=7)	p-value
ICSI (%)	17 (60.7)	55 (63.2)	32 (62.7)	NS^C^	4 (40.0)	NS^c^	32 (53.3)	39 (45.9)	19 (54.3)	NS^C^	2 (28.6)	NS^C^
Gonadotropin dose (IU)	2736.61±693.57	2327.04±822.55	1949.021658.48	0.036^a^; <0.001^b^	2172.51916.63	NS^d^	2622.0311364.50	2599.851835.11	2281.621677.04	<0.001^a^; NS^b^	22251531.51	NS^d^
Number of stimulation days	10.00±2.11	9.75±1.83	9.9812.01	NS^ab^	10.9012.08	NS^d^	10.0512.84	10.0511.98	9.9411.65	NS^ab^	10.2912.29	NS^d^
Number of follicles	6.17±2.89	14.36±9.39	23,8619,80	<0.001^ab^	30.0010.00	0.001^d^	5.0912.21	11.8418.51	17.95110.97	<0.001^ab^	20.00114.14	NS^d^
Number of COC	6.61±3.11	11.1516.44	15.9817.71	0.004^a^; <0.001^b^	19.90112.55	<0.001^d^	6.2316.38	11.2818.91	10.8917.06	<0.001^ab^	8.5718.54	NS^d^
Oocyte immaturity rate (%)	13.38±11.90	19.06±18.29	20.20116.89	NS^ab^	19,87116,46	NS^d^	20.60120.54	17.41119.88	25.55123.70	NS^ab^	47.05122.00	<0.001^d^
Number of 2PN oocytes	3.92±2.10	4.64±3.31	7.2414.59	NS^a^; <0.001^b^	9.5014,60	<0.001^d^	2.7612.49	5.4414.60	4.9414.06	<0.001^a^; 0.030^b^	3.1413.02	NS^d^
Fertilization rate (%)	55.04±22.90	47.26±26.86	45.27124.39	NS^ab^	55.45125,58	NS^d^	49.73128.17	53.00130.63	45.55126.59	NS^ab^	37.94126.95	NS^d^
Cleavage rate (%)	99.31±3.40	97.5418.69	97.8618.99	NS^ab^	99.3312,11	NS^d^	98.1518.36	95.88113.69	99.1413.75	NS^ab^	100.0010.00	NS^d^
Blastocyst rate (%)	7.98±14.61	10.06±17.81	16.82117.02	NS^ab^	12.501-	0.011^d^	8.08118.33	5.06111.10	2.2918.87	NS^ab^	0.0010.00	NS^d^
Number of obtained embryos	2.26±1.25	2.35±1.53	2.9212.15	NS^ab^	4.0012.16	0.004^d^	1.5510.98	2.7312.04	2.4511.79	<0.001^a^; 0.047^b^	2.0011.92	NS^d^
Number of IVF cycles with *freeze all* [n (%)]	11 (39.3)	39 (44,8)	37 (72.5)	0,002^c^	10 (100,00)	0.002^c^	10 (16.7)	43 (50.6)	16 (45.7)	< 0.001^c^	2 (28.6)	NS^C^
Number of criopreserved embryos	1.18±1.19	1,46±1,74	2.5812.47	NS^a^; 0.036^b^	3.90±2.,18	<0.001^d^	0.4410.97	2.0512.17	1.7012.12	<0.001^a^; 0.031^b^	1.7512.87	NS^d^
Positive βpHCG [n (%)]	9(32.1)	53 (60,9)	32 (62.7)	0.013^c^	6(66.7)	NS^C^	22 (36.7)	37 (43.5)	14 (40.0)	NS^C^	4(57.1)	NS^C^
Clinical pregnancy rate (%)	8 (28.6)	41 (47,1)	26 (51.0)	NS^C^	6 (60.0)	0.00^	18 (30.0)	25 (29.4)	9(25.7)	NS^C^	2 (28.6)	<0.001^c^
Cumulative clinical pregnancy (%)	8 (28.6)	42 (48,3)	26 (51.0)	NS^c^	6 (60.0)	NS^C^	18 (30.0)	27 (31.8)	9 (25.7)	NS^C^	2 (28.6)	NS^C^
LBR (%)	8 (28.6)	33 (37,9)	23 (45.1)	NS^C^	4 (40.0)	0.004^c^	9 (15.0)	23 (27.1)	6(17.1)	0.039^c^	1 (14.3)	<0.001^c^
CLBR (%)	8 (28.6)	34 (39,1)	23 (45,1)	NS^C^	4 (40,0)	NS^C^	9 (15.0)	25 (29.4)	6(17.1)	0.038^c^	1 (14.3)	NS^C^
Endometrial thickness on the day of the last monitoring (mm)	9.99±1.75	9.94±1.91	10.3412.58	NS^ab^	10.9212.14	NS^d^	9.5111.92	10.0311.92	10.1012.14	NS^ab^	10.6912.93	NS^d^
Endometrial thickness on oocyte pick-up day (mm)	11.02±2.42	11.1512.36	10.9612.44	NS^ab^	11.5011.75	NS^d^	9.3611.41	10.0412.18	10.9812.08	NS^ab^	10.0213.37	NS^d^

Data expressed as mean±standard deviation. *p*-value < 0.05 was considered statistically significant. ICSI - Intracytoplasmatic sperm injection; IU - International units; COC - cumulus-oocyte complexes; 2PN - two pronucleated; IVF - in vitro fertilization; βHCG - human chorionic gonadotropin hormone; LBR - live birth rate; CLBR - Cumulative live birth rate; Pc - Percentile; NS - not statistically different; a - Comparison between “low” and “high” (One Way ANOVA); b - Comparison between “moderate” and “high” (One Way ANOVA); c - Chi-square test; d - Comparison between “very high” and below “very high” (One Way ANOVA).

In the analysis of women over 35 years old, statistically significant differences were observed among all groups of AMH levels. Within this age range, women with elevated AMH levels achieved a higher number of embryos (*p*<0.001 and *p*=0.047), a greater number of cryopreserved embryos (*p*<0.001 and *p*=0.031), and a higher biochemical pregnancy rate compared to the other groups. In this age group, the LBR and CLBR decreased from the moderate AMH group to the high AMH group, with LBR dropping from 27.1% to 17.1% (*p*=0.039) and CLBR decreasing from 29.4% to 17.1% (*p*=0.038).

Some results from the 17 women who had AMH levels above the 95th percentile changed after detailed analysis by age. Besides observing a greater number of embryos obtained in younger women (4.00 *vs*. 2.00), this number was also higher compared to women with AMH levels below the 95th percentile (*p*=0.004) below 35 years old. This result is not observed in the group of women over 35 years old. Also, the clinical pregnancy rate, cumulative clinical pregnancy rate, LBR, and CLBR were markedly lower in women above 35 years old compared to younger ones. Specifically, in the group of women with high (above the 75th percentile) and very high (above the 95th percentile) AMH levels, the LBR was 45.1% and 40.0% below 35 years old and 17.1% and 14.3% above 35 years old, respectively. The statistical analysis of the remaining variables did not change after logistic regression. The remaining results are in Tables 4 e 5.

### According to BMI classes

The IVF/ICSI cycles (n=494) were divided into groups according to BMI classes (n=493). It was found that the duration of infertility tended to be higher in groups of women with higher BMI, with statistical significance observed when comparing normal weight (*p*<0.001) and overweight classes (*p*=0.018) with obese women (Table 6). The IVF cycle characteristics and outcomes are presented in Table 6. Women with higher BMI tended to require higher doses of gonadotropins compared to underweight and normal weight women (*p*=0.025), overweight women (*p*=0.004), and obese women (*p*<0.001), as well as between normal weight and obese women (*p*=0.041). Across BMI groups, there was a trend towards a greater number of stimulation days, with statistical significance between normal weight and overweight classes (*p*=0.003) and normal weight and obese classes (*p*<0.001). There was a significant increase in the number of COC (*p*=0.047), but a decrease in fertilization rate (*p*=0.020) in overweight women compared to underweight women. The remaining prognostic factors were not associated with significant differences between groups.

**Table 6:** Baseline reproductive characteristics and hormone levels of in vitro fertilization (IVF) cycles according to body mass index (BMI) classes (n=493).

	Underweight (<18.5 kg/m^2^) (n=23)	Normal weight (18.5-24.9 kg/m^2^) (n=285)	Overweight (25-29.9 kg/m^2^) (n=106)	Obesity (>30 kg/m^2^) (n=79)	p-value
Age (years)	35.43±3.37	34.90±3.34	34.43i3.78	34.71i4.00	NS^a.b.c.d.e.f^
Weight (Kg)	49.24±3.36	58.48±6.18	71.84i6.59	88.32ill.77	<0.001^abcdef^
Height (cm)	166.39±5.39	163.34i5.92	162.80i5.85	160.90i6.65	<0.001^c^; 0.008^e^; NS^abdf^
Primary infertility type [n (%)]	19 (82.6)	224(78.6)	81(76.4)	63(80.8)	NS^g^
Female smoker [n (%)]	6(75.0)	25(19.4)	7(15.9)	8(22.9)	0.020^g^
Male smoker [n (%)]	6(75.0)	44(34.6)	22(50.0)	15(41.7)	NS^g^
Couple smoker [n (%)]	6(75.0)	15(11.8)	5(11.4)	7(19.4)	< 0.001^g^
PCOS + other infertility factor [n (%)]	2(8.7)	25(8.8)	10 (9.4)	12(15.2)	NS^g^
Male infertility factor [n (%)]	17 (73.9)	186(65.3)	71(67.0)	53(67.1)	NS^g^
Duration of infertility (months)	46.87±23.10	47.18i27.03	51.90i30.81	65.84i47.91	<0.001^e^; 0.018^f^; NS^a bcd^
LH,mlU/mL	6.28±2.24	7.47±5.13	7.25±3.89	6.47±5.23	NS^a.b.c.d.e.f^
FSH,mlU/mL	6.94Ü.76	8.0Ü6.78	7.33±3.04	6.80±2.98	|NS^a.b.c.d.e.f^
LH:FSH ratio	0.93±0.32	1.00±0.50	1.05±0.65	1.08Ü.09	NS^a.b.c.d.e.f^
Basal estradiol, pg/mL	53.07±18.41	52.13i47.50	41.04i20.97	48.96i44.87	NS^a.b.c.d.e.f^
Progesterone, ng/mL	0.98±0.90	1.13±1.27	0.84±0.47	0.99±1.08	NS^a.b.c.d.e.f^

Data expressed as mean±standard deviation. *p*-value < 0.05 was considered statistically significant.; PCOS: polycystic ovary syndrome; LH: luteinizing hormone (at beginning of the menstrual cycle); FSH: follicle-stimulating hormone (at beginning of the menstrual cycle); IU - International units; NS - not statistically different; a - Comparison between underweight and normal weight (One Way ANOVA); b - Comparison between underweight and overweight (One Way ANOVA); c - Comparison between underweight and obesity (One Way ANOVA); d - Comparison between normal weight and overweight (One Way ANOVA); e - Comparison between normal weight and obesity (One Way ANOVA); f - Comparison between overweight and obesity (One Way ANOVA); g - Chi-square test.

Regarding LH levels, FSH levels, LH:FSH ratio, basal estradiol, and progesterone at trigger, no statistically significant differences were found between BMI classes. There is a decrease in the clinical pregnancy rate, cumulative pregnancy rate, LBR, and CLBR in the underweight and obese groups compared to the other BMI classes, with higher values observed in normal weight and overweight women. However, these findings are not statistically significant. The remaining results are in Tables 6 and 7

**Table 7:** In vitro fertilization (IVF) intra-cycle characteristics and assisted reproductive technology (ART) outcomes according to body mass index (BMI) classes (n=493).

	Underweight (<18.5 kg/m^2^) (n=23)	Normal weight (18.5-24.9 kg/m^2^) (n=285)	Overweight (25-29.9 kg/m^2^) (n=106)	Obesity (>30 kg/m^2^) (n=79)	p-value
ICSI (%)	9 (39.1)	147 (51.6)	57 (53.8)	44(55.7)	NS^g^
Gonadotropin dose (IU)	1672.83±949.43	2254.60±932.83	2418.99±920.25	2576.44±1040.16	0.025^a^; 0.004^b^; <0.001^c^; 0.041^e^; NS^d,f^
Number of stimulation days	9.00±2.13	9.4511.90	10.26±2.28	10.54±2.19	NS^a bc f^; 0.003^d^; <0.001^e^
Number of follicles	13.00110.74	12.9419.67	16.04110.73	15.15±11.10	NS^a.b.c.d.e.f^
Number of COC	8.0015.99	10.4617.83	12.5617.77	9.63±7.08	0.047^b^; NS^acdef^
Oocyte immaturity rate (%)	18.96122.18	16.86116.85	18.98118.03	19.07120.13	NS^a.b.c.d.e.f^
Number of 2PN oocytes	4.9114.06	4.8913.96	5.7014.15	4.84±4.04	NS^a.b.c.d.e.f^
Fertilization rate (%)	65.09130.40	50.00126.75	46.61126.61	52.60127.24	0.020^b^; NS^a c def^
Cleavage rate (%)	100.00	97.09110.67	97.8117.94	98.66	NS^a.b.c.d.e.f^
Blastocyst rate (%)	5.60	6.99	9.12	3.78	NS^a.b.c.d.e.f^
Number of obtained embryos	2.4312.57	2.4111.81	2.8312.08	2.4712.05	NS^a.b.c.d.e.f^
Number of IVF cycles with *freeze all* [n (%)]	8 (34.8)	113 (39.8)	53 (50.0)	34 (43.0)	NS^g^
Number of criopreserved embryos	1.8413.04	1.4111.95	1.8112.23	1.4212.09	NS^a.b.c.d.e.f^
Positive βHCG [n (%)]	9 (39.1)	140 (49.1)	57 (53.8)	38 (48.1)	NS^g^
Clinical pregnancy rate (%)	26.5	40.3	41.4	31.4	NS^g^
Cumulative clinical pregnancy (%)	29.4	44.0	52.2	35.7	NS^g^
LBR (%)	26.5	34.5	32.1	29.3	NS^g^
CLBR (%)	29.4	37.6	42.2	31.9	NS^g^
Endometrial thickness on the day of the last monitoring (mm)	9.6511.72	9.6311.93	10.0712.16	10.6012.23	NS^a.b.c.d.f^; 0.001^e^
Endometrial thickness on oocyte pick-up day (mm)	11.4712.72	10.2212.19	10.5612.00	11.1312.39	NS^a.b.c.d.e.f^

Data expressed as mean±standard deviation. *p*-value < 0.05 was considered statistically significant. ICSI - Intracytoplasmatic sperm injection; IU - International units; COC - cumulus-oocyte complexes; 2PN - two pronucleated; IVF - in vitro fertilization; βHCG - human chorionic gonadotropin hormone; LBR - live birth rate; CLBR - Cumulative live birth rate; NS - not statistically different; a - Comparison between underweight and normal weight (One Way ANOVA); b - Comparison between underweight and overweight (One Way ANOVA); c - Comparison between underweight and obesity (One Way ANOVA); d - Comparison between normal weight and overweight (One Way ANOVA); e - Comparison between normal weight and obesity (One Way ANOVA); f - Comparison between overweight and obesity (One Way ANOVA); g - Chi-square test.

### According to age

Splitting the sample into two groups below and above 35 years old (n=238 and n=256, respectively), it is observed that there are more women with primary infertility causes in the group below 35 years old (86.6% *vs*. 71.0%; *p*<0.001), the duration of this infertility is shorter (48.05 months *vs*. 54.04 months; *p*=0.041), and the LH:FSH ratio is lower in women above 35 years old (0.90 *vs*. 1.15; *p*<0.001) (Table 8).

**Table 8:** Baseline reproductive characteristics and hormone levels of in vitro fertilization (IVF) cycles according to age (n=494).

	< 35 years (n=238)	> 35 years (n=256)	p-value
Weight (Kg)	66.51±13.69	64.96±13.64	NS^b^
Height (cm)	162.92±6.04	163.01±6.16	NS^b^
BMI (Kg/m^2^)	25.05±4.90	24.5±5.32	NS^b^
Primary infertility type [n (%)]	206 (86.6)	181 (71.0)	<0.001^a^
Female smoker [n (%)]	20(20.6)	26 (21.7)	NS^a^
Male smoker [n (%)]	41 (42.7)	46 (38.3)	NS^a^
Couple smoker [n (%)]	17 (17.7)	16 (13.3)	NS^a^
PCOS + other infertility factor [n (%)]	20 (8.4)	29 (11.3)	NS^a^
Male infertility factor [n (%)]	186 (78.2)	142 (55.5)	<0.001^a^
Duration of infertility (months)	48.05±29.32	54.04±34.98	0.041^b^
LH,mlU/mL	7.44±4.14	6.98±5.35	NS^b^
FSH,mlU/mL	7.04±2.19	8.27±7.30	0.014^b^
LH:FSH ratio	1.15±0.82	0.90±0.41	<0.001
Basal estradiol, pg/mL	50.57±51.22	48.18±30.74	NS^b^
Progesterone, ng/mL	0.99±0.73	1.10±1.39	NS^b^

Data expressed as mean±standard deviation. *p*-value < 0.05 was considered statistically significant. BMI: body mass index; PCOS: polycystic ovary syndrome; LH: luteinizing hormone (at beginning of the menstrual cycle); FSH: follicle-stimulating hormone (at beginning of the menstrual cycle); IU - International units; NS - not statistically different; a - Chi-square test; b - One Way ANOVA.

The IVF cycle characteristics and outcomes are presented in Table 9. Regarding intra-cycle variables, younger women underwent more ICSI (59.2% *vs*. 45.7%; *p*=0.003) than FIV. The dose of gonadotropins used in stimulation was higher in older women (2472.63 IU *vs*. 2148.43 IU; *p*<0.001), while the number of COC (9.49 *vs*. 11.93; *p*<0.001), follicle count (11.40 *vs*. 16.30; *p*<0.001), number of embryos with 2PN (4.59 *vs*. 5.57; *p*=0.007), blastocyst rate (5.34% *vs*. 8.70%; *p*=0.032), number of freeze-all cycles (36.9% *vs*. 48.3%; *p*=0.010), biochemical pregnancy rate (41.8% *vs*. 58.0%; *p*<0.001), clinical pregnancy rate (29.9% *vs*. 47.4%; *p*<0.001), cumulative pregnancy rate (35.5% *vs*. 52.9%; *p*<0.001), LBR (23.7% *vs*. 42.5%; *p*<0.001), CLBR (28.4% *vs*. 47.1%; *p*<0.001), and endometrial thickness on oocyte pick-up day (10.06 mm *vs*. 11.02 mm; *p*=0.006) were lower in this group compared to women below 35 years old. The remaining results are in Tables 8 and 9.

**Table 9:** In vitro fertilization (IVF) intra-cycle characteristics and assisted reproductive technology (ART) outcomes according to age (n=494).

	< 35 years (n=238)	> 35 years (n=256)	p-value
ICSI (%)	141 (59.2)	117 (45.7)	0.003^a^
Gonadotropin dose (IU)	2148.43±797.01	2472.63il075.47	<0.001^b^
Number of stimulation days	9.76±1.90	9.80±2.27	NS^b^
Number of follicles	16.30110.51	ll.40i9.30	<0.001^b^
Number of COC	11.93±7.41	9.49±7.77	<0.001^b^
Oocyte immaturity rate (%)	17.14116.11	18.36il9.38	NS^b^
Number of 2PN oocytes	5.57±4.01	4.59±3.98	0.007^b^
Fertilization rate (%)	48.91±25.04	51.81i28.93	NS^b^
Cleavage rate (%)	98.0017.89	97.30il0.21	NS^b^
Blastocyst rate (%)	8.70115.34	5.34±13.01	0.032^b^
Number of obtained embryos	2.6511.98	2.40±1.94	NS^b^
Number of IVF cycles with *freeze all* [n (%)]	115 (48.3)	94 (36.9)	0.010^a^
Number of criopreserved embryos	1.6712.15	1.39±2.06	NS^b^
Positive βHCG [n (%)]	138 (58.0)	107 (41.8)	<0.001^a^
Clinical pregnancy rate (%)	47.4	29.9	<0.001^a^
Cumulative clinical pregnancy (%)	52.9	35.5	<0.001^a^
LBR(%)	42.5	23.7	<0.001^a^
CLBR (%)	47.1	28.4	<0.001^a^
Endometrial thickness on the day of the last monitoring (mm)	9.94±2.15	9.83±1.95	NS^b^
Endometrial thickness on oocyte pick-up day (mm)	11.0212.37	10.06i2.02	0.006^b^

Data expressed as mean±standard deviation. *p*-value < 0.05 was considered statistically significant. ICSI - Intracytoplasmatic sperm injection; IU - International units; COC - cumulus-oocyte complexes; 2PN - two pronucleated; IVF - in vitro fertilization; βHCG - human chorionic gonadotropin hormone; LBR - live birth rate; CLBR - Cumulative live birth rate; NS - not statistically different; a - Chi-square test; b - One Way ANOVA.

### According to LH:FSH ratio

Women with an LH:FSH ratio greater than 1 are significantly younger compared to women with a ratio lower than 1 (35.36 *vs*. 33.84 years; *p*<0.001). There is also a shorter duration of infertility in women with an LH:FSH ratio greater than 1 (54.2 *vs*. 46.0 months; *p*<0.008) (Table 10). The IVF cycle characteristics and outcomes are presented in Table 11. Regarding intra-cycle variables, in women with an LH:FSH ratio greater than 1, there is a reduction in the dose of gonadotropins used in stimulation (1921.25 IU *vs*. 2527.59 IU; *p*<0.001), a higher number of follicle count (18.59 *vs*. 11.36; *p*<0.001), a higher number of COC (13.06 *vs*. 9.45; *p*<0.001), a higher number of embryos with 2PN (6.11 *vs*. 4.52; *p*<0.001), a higher number of embryos obtained (2.87 *vs*. 2.34; *p*=0.006), a higher number of embryos cryopreserved (1.82 *vs*. 1.37; *p*=0.046), increased LBR (38.3% *vs*. 29.2%; *p*=0.007) and CLBR (45.2% *vs*. 32.5%; *p*=0.010), a higher number of freeze-all cycles (50.0% *vs*. 38.8%; *p*=0.017), and cases of biochemical pregnancy (59.6% *vs*. 43.6%; *p*=0.004). The remaining results are in Tables 10 and 11.

**Table 10:** Baseline reproductive characteristics and hormone levels of *in vitro* fertilization (IVF) cycles according to luteinizing hormone/follicle-stimulating hormone ratio (LH:FSH) (n=483).

	< 1.00 (n=305)	>1.00 (n=178)	p-value
Age (years)	35.36±3.29	33.84±3.79	<0.001^b^
Weight (Kg)	65.49±13.44	66.00±14.34	NS^b^
Height (cm)	163.16±5.96	162.50±6.34	NS^b^
BMI (Kg/m^2^)	24.61±4.94	25.03±5.48	NS^b^
Primary infertility type [n (%)]	234(77.0)	144 (80.9)	NS^a^
Female smoker [n (%)]	36 (24.2)	8(13.3)	0.030^a^
Male smoker [n (%)]	57 (38.5)	26(43.3)	NS^a^
Couple smoker [n (%)]	24 (16.2)	8 (13.3)	NS^a^
PCOS + other infertility factor [n (%)]	29 (9.5)	17(9.6)	NS^a^
Male infertility factor [n (%)]	195 (63.9)	124(69.7)	NS^a^
Duration of infertility (months)	54.17±33.94	45.96±29.93	0.008^b^
LH,mlU/mL	5.83±4.54	9.55±4.32	<0.001^b^
FSH,mlU/mL	8.34±6.71	6.50±.81	<0.001^b^
Basal estradiol basal, pg/mL	46.48±33.51	54.67±53.04	0.043^b^
Progesterone, ng/mL	0.99±1.17	1.30±0.92	NS^b^

Data expressed as mean±standard deviation. *p*-value < 0.05 was considered statistically significant. BMI: body mass index; PCOS: polycystic ovary syndrome; LH: luteinizing hormone (at beginning of the menstrual cycle); FSH: follicle-stimulating hormone (at beginning of the menstrual cycle); IU - International units; NS - not statistically different; a - Chi-square test; b - One Way ANOVA.

**Table 11:** In vitro fertilization (IVF) intra-cycle characteristics and assisted reproductive technology (ART) outcomes according to luteinizing hormone/follicle-stimulating hormone ratio (LH:FSH) (n=483).

	< 1.00 (n=305)	>1.00 (n=178)	p-value
ICSI (%)	168 (55.1)	84 (47.2)	NS^a^
Gonadotropin dose (IU)	2527.59±1016.55	1921.251728.23	<0.001^b^
Number of stimulation days	9.90±2.19	9.5611.92	NS^b^
Number of follicles	11.36±9.11	18.59110.47	<0.001^b^
Number of COC	9.4517.41	13.0617.75	<0.001^b^
Oocyte immaturity rate (%)	18.09118.61	16.78116.72	NS^b^
Number of 2PN oocytes	4.5213.79	6.1114.27	<0.001^b^
Fertilization rate (%)	50.79128.45	49.97124.99	NS^b^
Cleavage rate (%)	98.2317.34	96.56111.58	NS^b^
Blastocyst rate (%)	6.96115.24	6.22111.58	NS^b^
Number of obtained embryos	2.3411.80	2.8712.20	0.006^b^
Number of IVF cycles with *freeze all* [n (%)]	118(38.8)	89(50.0)	0.017^a^
Number of criopreserved embryos	1.3711.95	1.8212.33	0.046^b^
Positive βpHCG [n (%)]	133(43.6)	106(59.6)	0.004^a^
Clinical pregnancy rate (%)	36.5	41.6	NS^a^
Cumulative clinical pregnancy (%)	41.1	48.8	NS^a^
LBR (%)	29.2	38.3	0.007^a^
CLBR(%)	32.5	45.2	0.010^a^
Endometrial thickness on the day of the last monitoring (mm)	9.8512.06	9.9412.08	NS^b^
Endometrial thickness on oocyte pick-up day (mm)	10.2812.14	11.0312.50	NS^b^

Data expressed as mean±standard deviation. *p*-value < 0.05 was considered statistically significant. ICSI - Intracytoplasmatic sperm injection; IU - International units; COC - cumulus-oocyte complexes; 2PN - two pronucleated; IVF - in vitro fertilization; βHCG - human chorionic gonadotropin hormone; LBR - live birth rate; CLBR - Cumulative live birth rate; NS - not statistically different; a - Chi-square test; b - One Way ANOVA.

### Correlations between AMH and other variables

Statistically significant positive correlations were found between AMH and LH serum levels at the beginning of the menstrual cycle (*p*<0.001), the LH:FSH ratio (*p*<0.001), the number of COC (*p*<0.001) and follicle count (*p*<0.001), LBR (*p*=0.035), and CLBR (*p*=0.008). Negative correlations were observed between AMH serum levels and age (*p*<0.001), FSH levels (*p*<0.001), and the dose of gonadotropins used in stimulation (*p*<0.001). No statistically significant differences were found between AMH and BMI, basal estradiol, clinical pregnancy rate, and cumulative clinical pregnancy rate.

Regarding age, positive correlations were found with FSH levels at the beginning of the menstrual cycle (*p*=0.006) and the dose of gonadotropins used in stimulation (*p*<0.001), and negative correlations were found with LH levels at the beginning of the menstrual cycle (*p*<0.001), the LH:FSH ratio (*p*<0.001), the number of COC (*p*<0.001) and follicle count (*p*<0.001), clinical pregnancy rate (*p*<0.001), cumulative clinical pregnancy rate (*p*<0.001), LBR (*p*<0.001), and CLBR (*p*<0.001).

Regarding BMI, positive correlations were found between the dose of gonadotropins used in stimulation (*p*=0.003) and the number of follicle count (*p*=0.010), and negative correlations were found between LH and FSH levels at the beginning of the menstrual cycle (*p*<0.001 and *p*<0.001, respectively) and basal estradiol (*p*=0.003). The remaining variables showed no statistically significant correlations with BMI.

Positive correlations were observed between the LH:FSH ratio and LH levels at the beginning of the menstrual cycle (*p*<0.001), the number of COC (*p*<0.001) and follicle count (*p*<0.001), clinical pregnancy rate (*p*=0.030), cumulative clinical pregnancy rate (*p*=0.014), LBR (*p*=0.012), and CLBR (*p*=0.003). Negative correlations were found between the LH:FSH ratio and the dose of gonadotropins used in stimulation (*p*<0.001) and FSH levels at the beginning of the menstrual cycle (*p*<0.001). The remaining correlation coefficients are presented in Table 12.

**Table 12:** Spearman Correlations between anti-Müllerian hormone (AMH) levels, age, body mass index (BMI) and luteinizing hormone/follicle-stimulating hormone ratio (LH:FSH) and the *in vitro* fertilization (IVF) intra-cycle characteristics and assisted reproductive technology (ART) outcomes.

	AMH r	p-value	Age r	p-value	BMI r	p-value	LH:FSH ratio r	p-value
Age	-0.289	<0.001	-	___	-	___	-	-
BMI	0.041	NS	-0.040	NS	-	___	-	-
FSH	-0.359	<0.001	0.124	0.006	-0.135	0.003	-0.337	<0.001
LH	0.213	<0.001	-0.164	<0.001	-0.104	0.022	0.696	<0.001
LH:FSH ratio	0.510	<0.001	-0.272	<0.001	-0.019	NS	-	-
Basal estradiol	-0.051	NS	0.038	NS	-0.137	0.003	0.050	NS
Gonadotropin dose	-0.324	<0.001	0.241	<0.001	0.132	0.003	-0.388	<0.001
Number of COC	0.477	<0.001	-0.258	<0.001	0.037	NS	0.311	<0.001
Number of follicle	0.695	<0.001	-0.333	<0.001	0.135	0.010	0.459	<0.001
Clinical pregnancy rate	0.070	NS	-0.220	<0.001	-0.063	NS	0.107	0.030
Cumulative clinical pregnancy rate	0.099	NS	-0.215	<0.001	-0.036	NS	0.120	0.014
LBR	0.123	0.035	-0.236	<0.001	-0.052	NS	0.125	0.012
CLBR	0.153	0.008	-0.225	<0.001	-0.032	NS	0.144	0.003

BMI - Body mass index; FSH: follicle-stimulating hormone (at beginning of the menstrual cycle); LH: luteinizing hormone (at beginning of the menstrual cycle); COC - cumulus-oocyte complexes; LBR - live birth rate; CLBR - cumulative live birth rate; NS - not statistically different; r - Spearman’s correlation coefficient.

## DISCUSSION

The purpose of this research was to explore some basic reproductive characteristics, hormonal levels, and intra-cycle variables of IVF/ICSI associated to reproductive success, particularly LBR and CLBR, and consider their role in investigating reproductive prognosis in a sample of women with PCOS. Through comprehensive statistical analysis, the impact of these factors on reproductive outcomes was analyzed.

A weak negative correlation was found between AMH and age, supporting some descriptions in the literature indicating that this hormone tends to decrease with age (Erdem *et al*., 2002; Alsamarai *et al*., 2009; de Kat *et al*., 2016; Liu *et al*., 2022), although this correlation was not found in other studies (Tal *et al*., 2014; Vale-Fernandes *et al*., 2023). Regarding LH levels at the beginning of the menstrual cycle, they increase with AMH levels, as in the study by Liu *et al.* (2022). As for FSH levels, they were significantly lower in moderate AMH group, when comparing with the low AMH group, as described in other studies on the subject that have published data with samples of women with AMH levels close to those in this study (Liu *et al*., 2022; Wang *et al*., 2023). In this work, no differences were found between AMH groups and BMI, which may be explained by the fact that about 20% of women with PCOS are not obese (Casanova *et al*., 2019), supporting results already described (Sahmay *et al*., 2011; Liu *et al*., 2022; Vale-Fernandes *et al*., 2023). However, in women with very high AMH levels, above the 95th percentile (n=17), AMH was positively related to weight, the LH:FSH ratio, and BMI, consistent with some recent evidence (Lin *et al*., 2011). This conclusion may reinforce the well-established association between PCOS and conditions such as overweight, obesity, and increased fat mass, which affect approximately half of the women diagnosed with this endocrine disorder. This clinical relationship can be understood through the mechanisms that lead to dysregulation of the gonadotropin axis, particularly concerning hormones such as LH, androstenedione, estrone, testosterone, insulin, and appetite regulation (Casanova *et al*., 2019). The LH:FSH ratio significantly increased with AMH levels, supporting similar results in several studies (Casanova *et al*., 2019; Liu *et al*., 2022).

Additionally, the relationship between elevated AMH levels and higher progesterone levels at trigger can be discussed, which are associated with a higher number of IVF cycles with freeze all observed in this study. In fact, premature elevation of progesterone has implications for the decision of whether to perform embryo transfer or to freeze all embryos for subsequent FET, using the freeze-all method, as described in the literature (Healy *et al*., 2016; Wang *et al*., 2017). Premature elevation of progesterone appears to have a negative impact on implantation rates and pregnancy rates due to desynchronization with the endometrium (Aghahosseini *et al*., 2017).

Age-related variations show that women above 35 with high serum levels of AMH exhibit lower LBR and CLBR compared to younger women in the same percentile range of this hormone. This finding leads to the conclusion that AMH levels may contribute to LBR and CLBR, but age is also an important factor to consider. Specifically, in groups of women with elevated AMH (above the 75th percentile) and very high AMH (above the 95th percentile), the LBR was 45.1% and 40.0% below 35 years and 17.1% and 14.3% above 35 years, respectively. This result revealed that excessively high levels of AMH, as expected in a group of women with PCOS (Teede *et al*., 2018; Costello *et al*., 2019; Christ & Cedars, 2023), may contribute to a worse reproductive prognosis, as some studies have indicated (Lukaszuk *et al*., 2014; Vale-Fernandes *et al*., 2023), but age is an important factor to be considered. According to authors Blazar *et al.,* AMH level is related to reproductive outcome in the context of second-line infertility treatments, particularly in terms of pregnancy rate and LBR, regardless of age (Gleicher *et al*., 2010; Blazar *et al*., 2011), strengthening that AMH levels may carry greater significance than age. In women above 35, the biochemical pregnancy rate and cumulative pregnancy rate increased from low to moderate AMH level, but increases were no statistically significant. Also in women above 35, LBR and CLBR increased from low to moderate AMH levels but decreased by 10% (*p*=0.039) and 12.3% (*p*=0.038), respectively, from moderate to high AMH group, results that are opposite to some research on the subject (Tal *et al*., 2020; Liu *et al*., 2022; Vale-Fernandes *et al*., 2023), but consistent with other studies (te Velde & Pearson, 2002; Gnoth *et al*., 2003; van Rooij *et al*., 2005; Nelson *et al*., 2007; Gleicher *et al*., 2010; Blazar *et al*., 2011; La Marca *et al*., 2011; Brodin *et al*., 2013; Acharya *et al*., 2022; Wang *et al*., 2023; Delamuta *et al*., 2024). Acharya *et al.* conclude that women with AMH levels above 5 ng/mL (similar to the group of women in this study with AMH levels above 4.66 ng/mL), undergoing IVF with autologous fresh embryo transfer, experience a 3% decrease in the likelihood of achieving a live birth for each unit increase in AMH (Acharya *et al*., 2022).

Discrepancies may be attributed to differences in the populations of various studies and sample sizes, but also to inaccuracies in the definitions of LBR and CLBR. LBR is defined in most studies as the number of live births per woman in a group, however, there are articles that assume the number of births equals the number of live births (Olivius *et al*., 2002). Regarding CLBR, which is already a complex concept but suggested as an appropriate way to report the success of an IVF cycle encompassing fresh embryo transfer and subsequent FET (Germond *et al*., 2004), the definitions diverge even further. Defining the numerator and denominator of CLBR is a challenge and it is possible that this concept considers varying data across articles (Maheshwari *et al*., 2015). Some studies used the attainment of the first live birth as the numerator (Thurin-Kjellberg *et al*., 2009; Luke *et al*., 2012; Stern *et al*., 2013; Bodri *et al*., 2014), while others included all live births in a cycle (Li *et al*., 2014). As for the denominator, it can be the number of women who underwent ovarian stimulation or all those who underwent oocyte pick-up. In addition to the above, there is no established consensus on the interpretation of results in cases where women undergo a repeated IVF cycle after achieving a live birth, as some authors simply exclude subsequent cycles (Bodri *et al*., 2014) and others consider them as new cases (Gnoth *et al*., 2011). There are articles that define CLBR as the number of live births that occur in a group of women after embryo transfer and subsequent FET until all cryopreserved embryos are finished (Daya, 2005), as in this study, but others consider the number of live births that occur in a group of women after embryo transfer and subsequent FET until a predetermined number of IVF cycles (Li *et al*., 2014). These different methods of calculating CLBR, based on different assumptions, cannot be directly compared and discussed in this and other studies.

Although PCOS is associated with oligo-ovulatory cycles, these women typically respond favorably to ovarian stimulation for subsequent IVF procedures, achieving a higher number of follicles compared to the average of women undergoing the same treatments. However, they are associated with an unfavorable reproductive outcome compared to women with infertility due to other causes (Rajani *et al*., 2012; Piomboni *et al*., 2014; Yilmaz *et al*., 2016; Artimani *et al*., 2018). It is hypothesized that PCOS may be detrimental to the follicular microenvironment, disadvantaging optimal conditions for normal oocyte physiological functions (Palomba *et al*., 2017), their quality (Qiao & Feng, 2011), and endometrial competence (Piltonen, 2016). Regarding endometrial data in this study, it was found that endometrial thickness on the day of the last monitoring and on the day of oocyte pick-up did not show significant differences between groups. It is known that endometrial thickness influences implantation rates, although the exact appropriate thresholds are still not consensus. A study conducted in 2018 concluded that increasing endometrial thickness from 9mm to 16mm has a slight effect on increasing the probability of pregnancy, demonstrating this link (Bashiri *et al*., 2018; Liu *et al*., 2022), although it is not in line with the results of this research.

The dose of gonadotropins used in stimulation showed a tendency to decrease with increasing AMH levels, consistent with other studies suggesting that AMH may be useful in predicting ovarian response to gonadotropins, a mechanism possibly explained by decreased follicular sensitivity to FSH, premature follicle growth, and decreased estradiol (Gnoth *et al*., 2008). As expected, the number of antral follicles correlates positively (moderate correlation coefficient) with AMH levels, consistent with the results described in the study by Li *et al.* (2018). This correlation supports the assertion that AMH production by antral follicles is increased in PCOS, where the number of antral follicles is approximately doubled (Gnoth *et al*., 2008; Kaya *et al*., 2010). The number of embryos obtained decreased with AMH, contrary to described evidence (Delamuta *et al*., 2024). However, the oocyte immaturity rate, the fertilization rate and cleavage rate did not show statistically significant differences between groups, results consistent with other studies conducted in women with PCOS (Vale-Fernandes *et al*., 2023).

In this study, no significant differences were found between BMI classes regarding the number of antral follicles, which is also consistent with a study assessing the impact of BMI on reproductive success in women undergoing IVF techniques (Chaves *et al*., 2023). However, in this 2023 study, obese women obtained more mature oocytes compared to women with normal weight, a finding not observed in the present sample, where no significant differences were found between BMI classes regarding the rate of oocyte immaturity. There was a significant increase in fertilization rate in overweight women compared to those in the first BMI class studied (65.1% *vs*. 46.6%), which is also consistent with the results of other studies (Shah *et al*., 2011; Chaves *et al*., 2023). These data may be explained considering the failure of oocyte cytoplasm in transporting condensed chromosomes or in the incorporation of sperm into oocytes (Shah *et al*., 2011; Machtinger *et al*., 2012).

It is noted that the clinical pregnancy rate, cumulative clinical pregnancy rate, LBR and CLBR were lower in obese women compared to other BMI classes; however, this difference did not reach statistical significance as expected and as described in several studies (Shah *et al*., 2011; Bellver *et al*., 2013; Goldman *et al*., 2019; Moreira *et al*., 2025). The LBR was lower in women with obesity, although it was not statistically significant, but this trend is also found in a study that suggests that the impact of BMI is more significant in younger ages and that this LBR is more influenced by the prevalence of aneuploidies than by BMI (Goldman *et al*., 2019). The decrease in clinical pregnancy rate in that study was observed in cases of higher degrees of obesity (Shah *et al*., 2011), while in the present study, an analysis was only conducted with BMI above 30 kg/m^2^, representing a limitation.

A study comparing IVF outcomes between women below and above 35 years reported that, for younger women, there was no difference between BMI categories in the number of mature oocytes, number of embryos obtained, fertilization rates, and clinical pregnancy rates (Shah *et al*., 2011; Vural *et al*., 2016). Except for the clinical pregnancy rate, which was significantly lower in women above 35 years old in this sample, all other factors were consistent with the results of this cited study. The authors Vural *et al.,* describe that in the group of women above 35 years old, the clinical pregnancy rate was lower in the obesity group, a result that is in line with this research, where obese women had a clinical pregnancy rate of 31.4% compared to clinical pregnancy rates in the normal weight and overweight classes of 40.3% and 41.4%, respectively (Vural *et al*., 2016). However, the number of mature oocytes, embryos obtained, and fertilization rate did not differ between BMI categories. Thus, Vural *et al.* suggest that different stages of IVF may be affected by the age of the woman undergoing treatment (Vural *et al*., 2016). The embryo cleavage rate did not show statistically significant differences between BMI classes, but there is a study reporting that embryos from women with obesity experience a delay in cleavage compared to normal-weight women, suggesting that there is slower embryonic development associated with higher maternal BMI (Bartolacci *et al*., 2019).

The hypersecretion of LH appears to impact fertility, as well as reproductive outcomes in women with PCOS. A high LH:FSH ratio is associated with poorer outcomes in terms of pregnancy and LBR, and in this study, a reduction in LBR was observed when this ratio was above 1. This finding is consistent with a study that evaluated the effect of this ratio in a sample of women with PCOS and found no statistically significant relationship with the number of embryos obtained and clinical pregnancy rate (Taylor *et al*., 1997). Furthermore, another article describes that in women with PCOS undergoing IVF techniques, an LH:FSH ratio greater than 1.5 decreased the clinical pregnancy rate (Wiser *et al*., 2013). In the present investigation, differences were found in the fertilization rate, which was lower when the LH:FSH ratio was higher, contrary to the study by Taylor *et al.*, where no differences were found in dividing samples into ratios greater or less than 1 (Taylor *et al*., 1997). Positive correlations were found between the LH:FSH ratio and LBR and CLBR, opposite to what Singh *et al.,* and other authors describe: a high LH:FSH ratio does not impair the outcome of IVF/ICSI cycles in women with PCOS (Ganor-Paz *et al*., 2016; Singh *et al*., 2021). It is thought that the deleterious effects of high LH may be potentially greater in women with PCOS with high LH:FSH ratios. In a 2012 study, the impact of this elevated ratio on reproductive outcomes in IVF treatments was evaluated, associating that a GnRH agonist protocol likely induces a sustained reduction in LH levels, affecting oocyte quality and implantation potential (Orvieto *et al*., 2012).

This study had some limitations such as its retrospective nature, which, for example, did not allow for the reassessment of anthropometric data during treatment, making it impossible to detect changes in BMI during this period. Additionally, BMI is a nonspecific marker of body composition, failing to distinguish between lean mass and fat mass. The differing definitions of CLBR from article to article made it difficult to compare existing studies with the data obtained in this investigation.

## CONCLUSIONS

AMH measurement supports clinical decision-making but should not be considered an isolate predictor of reproductive success in IVF techniques. Due to some conflicting data, there remains an urgent need to further investigate the effect of this hormone, exploring both existing and emerging hypotheses, not only as a marker of ovarian reserve. Age has a greater impact on LBR than BMI.

Couples facing infertility primarily seek to understand their chances of achieving a live birth before embarking on ART treatments, whether they involve fresh embryo transfers, the use of any surplus frozen embryos from their initial attempt, or subsequent additional treatments. Therefore, in practice, CLBR is the most useful metric as it summarizes the likelihood of a couple achieving a live birth over the entire treatment period. However, although CLBR can be communicated in research articles, due to the use of varied definitions based on different assumptions, it is necessary to standardize its definition and values to accurately reflect the success rate of IVF/ICSI.

## References

[r1] Acharya KS, Harris BS, Weber JM, Truong T, Pieper C, Eaton JL. (2022). Impact of increasing antimüllerian hormone level on in vitro fertilization fresh transfer and live birth rate. F S Rep.

[r2] Aghahosseini M, Aleyasin A, Sarfjoo FS, Mahdavi A, Yaraghi M, Saeedabadi H. (2017). In vitro fertilization outcome in frozen versus fresh embryo transfer in women with elevated progesterone level on the day of HCG injection: An RCT. Int J Reprod Biomed.

[r3] Alsamarai S, Adams JM, Murphy MK, Post MD, Hayden DL, Hall JE, Welt CK. (2009). Criteria for polycystic ovarian morphology in polycystic ovary syndrome as a function of age. J Clin Endocrinol Metab.

[r4] Artimani T, Karimi J, Mehdizadeh M, Yavangi M, Khanlarzadeh E, Ghorbani M, Asadi S, Kheiripour N. (2018). Evaluation of pro-oxidant-antioxidant balance (PAB) and its association with inflammatory cytokines in polycystic ovary syndrome (PCOS). Gynecol Endocrinol.

[r5] Bartolacci A, Buratini J, Moutier C, Guglielmo MC, Novara PV, Brambillasca F, Renzini MM, Dal Canto M. (2019). Maternal body mass index affects embryo morphokinetics: a time-lapse study. J Assist Reprod Genet.

[r6] Bashiri A, Halper KI, Orvieto R. (2018). Recurrent Implantation Failure-update overview on etiology, diagnosis, treatment and future directions. Reprod Biol Endocrinol.

[r7] Bellver J, Pellicer A, García-Velasco JA, Ballesteros A, Remohí J, Meseguer M. (2013). Obesity reduces uterine receptivity: clinical experience from 9,587 first cycles of ovum donation with normal weight donors. Fertil Steril.

[r8] Blazar AS, Lambert-Messerlian G, Hackett R, Krotz S, Carson SA, Robins JC. (2011). Use of in-cycle antimüllerian hormone levels to predict cycle outcome. Am J Obstet Gynecol.

[r9] Bodri D, Kawachiya S, De Brucker M, Tournaye H, Kondo M, Kato R, Matsumoto T. (2014). Cumulative success rates following mild IVF in unselected infertile patients: a 3-year, single-centre cohort study. Reprod Biomed Online.

[r10] Brodin T, Hadziosmanovic N, Berglund L, Olovsson M, Holte J. (2013). Antimüllerian hormone levels are strongly associated with live-birth rates after assisted reproduction. J Clin Endocrinol Metab.

[r11] Bull JR, Rowland SP, Scherwitzl EB, Scherwitzl R, Danielsson KG, Harper J. (2019). Real-world menstrual cycle characteristics of more than 600,000 menstrual cycles. NPJ Digit Med.

[r12] Burger HG. (2002). Androgen production in women. Fertil Steril.

[r13] Carmina E, Azziz R, Bergfeld W, Escobar-Morreale HF, Futterweit W, Huddleston H, Lobo R, Olsen E. (2019). Female Pattern Hair Loss and Androgen Excess: A Report From the Multidisciplinary Androgen Excess and PCOS Committee. J Clin Endocrinol Metab.

[r14] Casanova R, Chuang A, Goepfert AR, Hueppchenn NA, Weiss PM, Beckman CRB, Ling FW, Herbert WMP, Laube DW, Smith RP., Casanova R, Chuang A, Goepfert AR, Hueppchenn NA, Weiss PM, Beckman CRB, Ling FW, Herbert WMP, Laube DW, Smith RP. (2019). Beckmann and Ling’s Obstetrics and Gynecology.

[r15] Chang HM, Zhu YM, Leung PCK., Leung PCK, Adashi EY (2019). The Ovary.

[r16] Chaves HL, Schmitz MJ, Francisquini CDS, Rosa VBD, Schuffner A, Furlan JA. (2023). Impact of Body Mass Index and advanced maternal age on in vitro fertilization outcomes. JBRA Assist Reprod.

[r17] Christ JP, Cedars MI. (2023). Current Guidelines for Diagnosing PCOS. Diagnostics.

[r18] Christ JP, Gunning MN, Meun C, Eijkemans MJC, van Rijn BB, Bonsel GJ, Laven JSE, Fauser BCJM. (2019). Pre-Conception Characteristics Predict Obstetrical and Neonatal Outcomes in Women With Polycystic Ovary Syndrome. J Clin Endocrinol Metab.

[r19] Clark NM, Podolski AJ, Brooks ED, Chizen DR, Pierson RA, Lehotay DC, Lujan ME. (2014). Prevalence of Polycystic Ovary Syndrome Phenotypes Using Updated Criteria for Polycystic Ovarian Morphology: An Assessment of Over 100 Consecutive Women Self-reporting Features of Polycystic Ovary Syndrome. Reprod Sci.

[r20] Cook CL, Siow Y, Brenner AG, Fallat ME. (2002). Relationship between serum müllerian-inhibiting substance and other reproductive hormones in untreated women with polycystic ovary syndrome and normal women. Fertil Steril.

[r21] Costello MF, Garad RM, Hart R, Homer H, Johnson L, Jordan C, Mocanu E, Qiao J, Rombauts L, Teede HJ, Vanky E, Venetis CA, Ledger WL. (2019). A Review of Secondand Third-line Infertility Treatments and Supporting Evidence in Women with Polycystic Ovary Syndrome. Med Sci (Basel).

[r22] Cunha A, Póvoa AM. (2021). Infertility management in women with polycystic ovary syndrome: a review. Porto Biomed J.

[r23] Das M, Gillott DJ, Saridogan E, Djahanbakhch O. (2008). Anti-Mullerian hormone is increased in follicular fluid from unstimulated ovaries in women with polycystic ovary syndrome. Hum Reprod.

[r24] Daya S. (2005). Life table (survival) analysis to generate cumulative pregnancy rates in assisted reproduction: are we overestimating our success rates?. Hum Reprod.

[r25] de Kat AC, van der Schouw YT, Eijkemans MJ, Herber-Gast GC, Visser JA, Verschuren WM, Broekmans FJ. (2016). Back to the basics of ovarian aging: a population-based study on longitudinal anti-Müllerian hormone decline. BMC Med.

[r26] Delamuta LC, Fassolas G, Dias JA, Henrique LFO, Izzo FPM, Izzo CR. (2024). Antimüllerian hormone levels and IVF outcomes in polycystic ovary syndrome women: a scoping review. JBRA Assist Reprod.

[r27] DeUgarte CM, Woods KS, Bartolucci AA, Azziz R. (2006). Degree of facial and body terminal hair growth in unselected black and white women: toward a populational definition of hirsutism. J Clin Endocrinol Metab.

[r28] Diamanti-Kandarakis E, Dunaif A. (2012). Insulin resistance and the polycystic ovary syndrome revisited: an update on mechanisms and implications. Endocr Rev.

[r29] Dumont A, Robin G, Catteau-Jonard S, Dewailly D. (2015). Role of Anti-Müllerian Hormone in pathophysiology, diagnosis and treatment of Polycystic Ovary Syndrome: a review. Reprod Biol Endocrinol.

[r30] Eldar-Geva T, Ben-Chetrit A, Spitz IM, Rabinowitz R, Markowitz E, Mimoni T, Gal M, Zylber-Haran E, Margalioth EJ. (2005). Dynamic assays of inhibin B, anti-Mullerian hormone and estradiol following FSH stimulation and ovarian ultrasonography as predictors of IVF outcome. Hum Reprod.

[r31] Elgindy EA, El-Haieg DO, El-Sebaey A. (2008). Anti-Müllerian hormone: correlation of early follicular, ovulatory and midluteal levels with ovarian response and cycle outcome in intracytoplasmic sperm injection patients. Fertil Steril.

[r32] Erdem A, Erdem M, Biberoglu K, Hayit O, Arslan M, Gursoy R. (2002). Age-related changes in ovarian volume, antral follicle counts and basal FSH in women with normal reproductive health. J Reprod Med.

[r33] Escobar-Morreale HF, Carmina E, Dewailly D, Gambineri A, Kelestimur F, Moghetti P, Pugeat M, Qiao J, Wijeyaratne CN, Witchel SF, Norman RJ. (2012). Epidemiology, diagnosis and management of hirsutism: a consensus statement by the Androgen Excess and Polycystic Ovary Syndrome Society. Hum Reprod Update.

[r34] Fallat ME, Siow Y, Marra M, Cook C, Carrillo A. (1997). Müllerian-inhibiting substance in follicular fluid and serum: a comparison of patients with tubal factor infertility, polycystic ovary syndrome, and endometriosis. Fertil Steril.

[r35] Ganor-Paz Y, Friedler-Mashiach Y, Ghetler Y, Hershko-Klement A, Berkovitz A, Gonen O, Shulman A, Wiser A. (2016). What is the best treatment for women with polycystic ovarian syndrome and high LH/FSH ratio? A comparison among in vitro fertilization with GnRH agonist, GnRH antagonist and in vitro maturation. J Endocrinol Invest.

[r36] Gao H, Liu DE, Li Y, Wu X, Tan H. (2021). Early prediction of live birth for assisted reproductive technology patients: a convenient and practical prediction model. Sci Rep.

[r37] Germond M, Urner F, Chanson A, Primi MP, Wirthner D, Senn A. (2004). What is the most relevant standard of success in assisted reproduction?: The cumulated singleton/twin delivery rates per oocyte pick-up: the CUSIDERA and CUTWIDERA. Hum Reprod.

[r38] Gleicher N, Weghofer A, Barad DH. (2010). Anti-Müllerian hormone (AMH) defines, independent of age, low versus good live-birth chances in women with severely diminished ovarian reserve. Fertil Steril.

[r39] Gnoth C, Godehardt D, Godehardt E, Frank-Herrmann P, Freundl G. (2003). Time to pregnancy: results of the German prospective study and impact on the management of infertility. Hum Reprod.

[r40] Gnoth C, Maxrath B, Skonieczny T, Friol K, Godehardt E, Tigges J. (2011). Final ART success rates: a 10 years survey. Hum Reprod.

[r41] Gnoth C, Schuring AN, Friol K, Tigges J, Mallmann P, Godehardt E. (2008). Relevance of anti-Mullerian hormone measurement in a routine IVF program. Hum Reprod.

[r42] Goldman RH, Farland LV, Thomas AM, Zera CA, Ginsburg ES. (2019). The combined impact of maternal age and body mass index on cumulative live birth following in vitro fertilization. Am J Obstet Gynecol.

[r43] Hazout A, Bouchard P, Seifer DB, Aussage P, Junca AM, Cohen-Bacrie P. (2004). Serum antimüllerian hormone/müllerian-inhibiting substance appears to be a more discriminatory marker of assisted reproductive technology outcome than follicle-stimulating hormone, inhibin B, or estradiol. Fertil Steril.

[r44] Healy MW, Patounakis G, Connell MT, Devine K, DeCherney AH, Levy MJ, Hill MJ. (2016). Does a frozen embryo transfer ameliorate the effect of elevated progesterone seen in fresh transfer cycles?. Fertil Steril.

[r45] Joham AE, Norman RJ, Stener-Victorin E, Legro RS, Franks S, Moran LJ, Boyle J, Teede HJ. (2022). Polycystic ovary syndrome. Lancet Diabetes Endocrinol.

[r46] Kaya C, Pabuccu R, Satıroglu H. (2010). Serum antimüllerian hormone concentrations on day 3 of the in vitro fertilization stimulation cycle are predictive of the fertilization, implantation, and pregnancy in polycystic ovary syndrome patients undergoing assisted reproduction. Fertil Steril.

[r47] La Marca A, Orvieto R, Giulini S, Jasonni VM, Volpe A, De Leo V. (2004a). Mullerian-inhibiting substance in women with polycystic ovary syndrome: relationship with hormonal and metabolic characteristics. Fertil Steril.

[r48] La Marca A, Malmusi S, Giulini S, Tamaro LF, Orvieto R, Levratti P, Volpe A. (2004b). Anti-Müllerian hormone plasma levels in spontaneous menstrual cycle and during treatment with FSH to induce ovulation. Hum Reprod.

[r49] La Marca A, Sighinolfi G, Radi D, Argento C, Baraldi E, Artenisio AC, Stabile G, Volpe A. (2010). Anti-Mullerian hormone (AMH) as a predictive marker in assisted reproductive technology (ART). Hum Reprod Update.

[r50] La Marca A, Nelson SM, Sighinolfi G, Manno M, Baraldi E, Roli L, Xella S, Marsella T, Tagliasacchi D, D’Amico R, Volpe A. (2011). Anti-Müllerian hormone-based prediction model for a live birth in assisted reproduction. Reprod Biomed Online.

[r51] Legro RS, Arslanian SA, Ehrmann DA, Hoeger KM, Murad MH, Pasquali R, Welt CK, Endocrine Society (2013). Diagnosis and treatment of polycystic ovary syndrome: an Endocrine Society clinical practice guideline. J Clin Endocrinol Metab.

[r52] Lekamge DN, Barry M, Kolo M, Lane M, Gilchrist RB, Tremellen KP. (2007). Anti-Müllerian hormone as a predictor of IVF outcome. Reprod Biomed Online.

[r53] Li HW, Lee VC, Lau EY, Yeung WS, Ho PC, Ng EH. (2014). Cumulative live-birth rate in women with polycystic ovary syndrome or isolated polycystic ovaries undergoing in-vitro fertilisation treatment. J Assist Reprod Genet.

[r54] Li Y, Tan JQ, Mai ZY, Yang DZ. (2018). Value of anti-Müllerian hormone in predicting pregnant outcomes of polycystic ovary syndrome patients undergone assisted reproductive technology. Zhonghua Fu Chan Ke Za Zhi.

[r55] Lin YH, Chiu WC, Wu CH, Tzeng CR, Hsu CS, Hsu MI. (2011). Antimüllerian hormone and polycystic ovary syndrome. Fertil Steril.

[r56] Liu S, Hong L, Mo M, Xiao S, Wang X, Fan X, Zhang S, Diao L, Zeng Y. (2022). Association of antimüllerian hormone with polycystic ovarian syndrome phenotypes and pregnancy outcomes of in vitro fertilization cycles with fresh embryo transfer. BMC Pregnancy Childbirth.

[r57] Longcope C. (1986). Adrenal and gonadal androgen secretion in normal females. Clin Endocrinol Metab.

[r58] Lukaszuk K, Liss J, Kunicki M, Jakiel G, Wasniewski T, Woclawek-Potocka I, Pastuszek E. (2014). Anti-Müllerian hormone (AMH) is a strong predictor of live birth in women undergoing assisted reproductive technology. Reprod Biol.

[r59] Luke B, Brown MB, Wantman E, Lederman A, Gibbons W, Schattman GL, Lobo RA, Leach RE, Stern JE. (2012). Cumulative birth rates with linked assisted reproductive technology cycles. N Engl J Med.

[r60] Machtinger R, Combelles CM, Missmer SA, Correia KF, Fox JH, Racowsky C. (2012). The association between severe obesity and characteristics of failed fertilized oocytes. Hum Reprod.

[r61] Maheshwari A, McLernon D, Bhattacharya S. (2015). Cumulative live birth rate: time for a consensus?. Hum Reprod.

[r62] Meczekalski B, Czyzyk A, Kunicki M, Podfigurna-Stopa A, Plociennik L, Jakiel G, Maciejewska-Jeske M, Lukaszuk K. (2016a). Fertility in women of late reproductive age: the role of serum anti-Müllerian hormone (AMH) levels in its assessment. J Endocrinol Invest.

[r63] Meczekalski B, Czyzyk A, Kunicki M, Podfigurna-Stopa A, Plociennik L, Jakiel G, Maciejewska-Jeske M, Lukaszuk K. (2016b). Erratum to: Fertility in women of late reproductive age: the role of serum anti-Müllerian hormone (AMH) levels in its assessment. J Endocrinol Invest.

[r64] Moran LJ, Misso ML, Wild RA, Norman RJ. (2010). Impaired glucose tolerance, type 2 diabetes and metabolic syndrome in polycystic ovary syndrome: a systematic review and meta-analysis. Hum Reprod Update.

[r65] Moreira T, Leal C, Barreiro M, Tomé A, Vale-Fernandes E. (2025). Predictors of Pregnancy after Artificial Insemination in Women with Polycystic Ovary Syndrome. JBRA Assist Reprod.

[r66] Nardo LG, Yates AP, Roberts SA, Pemberton P, Laing I. (2009). The relationships between AMH, androgens, insulin resistance and basal ovarian follicular status in non-obese subfertile women with and without polycystic ovary syndrome. Hum Reprod.

[r67] National Institutes of Health (NIH) (2012). Evidence-based methodology workshop on polycystic ovary syndrome (PCOS).

[r68] Nelson SM, Yates RW, Fleming R. (2007). Serum anti-Müllerian hormone and FSH: prediction of live birth and extremes of response in stimulated cycles--implications for individualization of therapy. Hum Reprod.

[r69] Olivius K, Friden B, Lundin K, Bergh C. (2002). Cumulative probability of live birth after three in vitro fertilization/intracytoplasmic sperm injection cycles. Fertil Steril.

[r70] Orvieto R, Meltcer S, Liberty G, Rabinson J, Anteby EY, Nahum R. (2012). Does day-3 LH/FSH ratio influence in vitro fertilization outcome in PCOS patients undergoing controlled ovarian hyperstimulation with different GnRH-analogue?. Gynecol Endocrinol.

[r71] Palomba S, Daolio J, La Sala GB. (2017). Oocyte Competence in Women with Polycystic Ovary Syndrome. Trends Endocrinol Metab.

[r72] Piltonen TT. (2016). Polycystic ovary syndrome: Endometrial markers. Best Pract Res Clin Obstet Gynaecol.

[r73] Piomboni P, Focarelli R, Capaldo A, Stendardi A, Cappelli V, Cianci A, La Marca A, Luddi A, De Leo V. (2014). Protein modification as oxidative stress marker in follicular fluid from women with polycystic ovary syndrome: the effect of inositol and metformin. J Assist Reprod Genet.

[r74] Piouka A, Farmakiotis D, Katsikis I, Macut D, Gerou S, Panidis D. (2009). Anti-Mullerian hormone levels reflect severity of PCOS but are negatively influenced by obesity: relationship with increased luteinizing hormone levels. Am J Physiol Endocrinol Metab.

[r75] Prior JC, Naess M, Langhammer A, Forsmo S. (2015). Ovulation Prevalence in Women with Spontaneous Normal-Length Menstrual Cycles - A Population-Based Cohort from HUNT3, Norway. PLoS One.

[r76] Qiao J, Feng HL. (2011). Extraand intra-ovarian factors in polycystic ovary syndrome: impact on oocyte maturation and embryo developmental competence. Hum Reprod Update.

[r77] Rajani S, Chattopadhyay R, Goswami SK, Ghosh S, Sharma S, Chakravarty B. (2012). Assessment of oocyte quality in polycystic ovarian syndrome and endometriosis by spindle imaging and reactive oxygen species levels in follicular fluid and its relationship with IVF-ET outcome. J Hum Reprod Sci.

[r78] Reed BG, Carr BR., Feingold KR, Ahmed SF, Anawalt B, Blackman MR, Boyce A, Chrousos G, Corpas E, de Herder WW, Dhatariya K, Dungan K, Hofland J, Kalra S, Kaltsas G, Kapoor N, Koch C, Kopp P, Korbonits M, Kovacs CS, Kuohung W, Laferrère B (2000). Endotext.

[r79] Sahmay S, Guralp O, Senturk LM, Imamoglu M, Kucuk M, Irez T. (2011). Serum anti-müllerian hormone concentrations in reproductive age women with and without polycystic ovary syndrome: the influence of body mass index. Reprod Med Biol.

[r80] Shah DK, Missmer SA, Berry KF, Racowsky C, Ginsburg ES. (2011). Effect of obesity on oocyte and embryo quality in women undergoing in vitro fertilization. Obstet Gynecol.

[r81] Singh N, Mishra N, Dogra Y. (2021). Do basal Luteinizing Hormone and Luteinizing Hormone/Follicle-Stimulating Hormone Ratio Have Significance in Prognosticating the Outcome of In vitro Fertilization Cycles in Polycystic Ovary Syndrome?. J Hum Reprod Sci.

[r82] Slayden SM, Moran C, Sams WM (2001). Boots LR, Azziz R. Hyperandrogenemia in patients presenting with acne. Fertil Steril.

[r83] Solomon CG, Hu FB, Dunaif A, Rich-Edwards JE, Stampfer MJ, Willett WC, Speizer FE, Manson JE. (2002). Menstrual cycle irregularity and risk for future cardiovascular disease. J Clin Endocrinol Metab.

[r84] Starace M, Orlando G, Alessandrini A, Piraccini BM. (2020). Female Androgenetic Alopecia: An Update on Diagnosis and Management. Am J Clin Dermatol.

[r85] Stern JE, Brown MB, Wantman E, Kalra SK, Luke B. (2013). Live birth rates and birth outcomes by diagnosis using linked cycles from the SART CORS database. J Assist Reprod Genet.

[r86] Swanson M, Sauerbrei EE, Cooperberg PL. (1981). Medical implications of ultrasonically detected polycystic ovaries. J Clin Ultrasound.

[r87] Tal R, Seifer CM, Khanimov M, Seifer DB, Tal O. (2020). High serum Antimullerian hormone levels are associated with lower live birth rates in women with polycystic ovarian syndrome undergoing assisted reproductive technology. Reprod Biol Endocrinol.

[r88] Tal R, Seifer DB, Khanimov M, Malter HE, Grazi RV, Leader B. (2014). Characterization of women with elevated antimüllerian hormone levels (AMH): correlation of AMH with polycystic ovarian syndrome phenotypes and assisted reproductive technology outcomes. Am J Obstet Gynecol.

[r89] Taylor AE, McCourt B, Martin KA, Anderson EJ, Adams JM, Schoenfeld D, Hall JE. (1997). Determinants of abnormal gonadotropin secretion in clinically defined women with polycystic ovary syndrome. J Clin Endocrinol Metab.

[r90] te Velde ER, Pearson PL. (2002). The variability of female reproductive ageing. Hum Reprod Update.

[r91] Teede HJ, Misso ML, Costello MF, Dokras A, Laven J, Moran L, Piltonen T, Norman RJ, International PCOS Network (2018). Recommendations from the international evidence-based guideline for the assessment and management of polycystic ovary syndrome. Hum Reprod.

[r92] Thurin-Kjellberg A, Olivius C, Bergh C. (2009). Cumulative live-birth rates in a trial of single-embryo or double-embryo transfer. N Engl J Med.

[r93] Tsakos E, Tolikas A, Daniilidis A, Asimakopoulos B. (2014). Predictive value of anti-müllerian hormone, follicle-stimulating hormone and antral follicle count on the outcome of ovarian stimulation in women following GnRH-antagonist protocol for IVF/ET. Arch Gynecol Obstet.

[r94] Vale-Fernandes E, Barreiro M, Leal C, Macedo RZ, Tomé A, Monteiro MP. (2023). Elevated Anti-Müllerian Hormone as a Prognostic Factor for Poor Outcomes of In Vitro Fertilization in Women with Polycystic Ovary Syndrome. Biomedicines.

[r95] van Keizerswaard J, Dietz de Loos ALP, Louwers YV, Laven JSE. (2022). Changes in individual polycystic ovary syndrome phenotypical characteristics over time: a long-term follow-up study. Fertil Steril.

[r96] van Rooij IA, Broekmans FJ, Scheffer GJ, Looman CW, Habbema JD, de Jong FH, Fauser BJ, Themmen AP, te Velde ER (2005). Serum antimullerian hormone levels best reflect the reproductive decline with age in normal women with proven fertility: a longitudinal study. Fertil Steril.

[r97] van Rooij IA, Broekmans FJ, te Velde ER, Fauser BC, Bancsi LF, de Jong FH, Themmen AP (2002). Serum anti-Müllerian hormone levels: a novel measure of ovarian reserve. Hum Reprod.

[r98] Vural F, Vural B, Çakiroglu Y. (2016). In vitro fertilization outcomes in obese women under and above 35 years of age. Clin Exp Obstet Gynecol.

[r99] Wang A, Santistevan A, Hunter Cohn K, Copperman A, Nulsen J, Miller BT, Widra E, Westphal LM, Yurttas Beim P. (2017). Freeze-only versus fresh embryo transfer in a multicenter matched cohort study: contribution of progesterone and maternal age to success rates. Fertil Steril.

[r100] Wang Q, Qi D, Zhang L, Wang J, Du Y, Lv H, Yan L. (2023). Association of the Cumulative Live Birth Rate with the Factors in Assisted Reproductive Technology: A Retrospective Study of 16,583 Women. J Clin Med.

[r101] Wild RA, Rizzo M, Clifton S, Carmina E. (2011). Lipid levels in polycystic ovary syndrome: systematic review and meta-analysis. Fertil Steril.

[r102] Wiser A, Shehata F, Holzer H, Hyman JH, Shalom-Paz E, Son WY, Tulandi T. (2013). Effect of high LH/FSH ratio on women with polycystic ovary syndrome undergoing in vitro maturation treatment. J Reprod Med.

[r103] Yilmaz N, Inal HA, Gorkem U, Sargin Oruc A, Yilmaz S, Turkkani A. (2016). Follicular fluid total antioxidant capacity levels in PCOS. J Obstet Gynaecol.

